# Mechanism of SARS-CoV-2 polymerase stalling by remdesivir

**DOI:** 10.1038/s41467-020-20542-0

**Published:** 2021-01-12

**Authors:** Goran Kokic, Hauke S. Hillen, Dimitry Tegunov, Christian Dienemann, Florian Seitz, Jana Schmitzova, Lucas Farnung, Aaron Siewert, Claudia Höbartner, Patrick Cramer

**Affiliations:** 1 Max Planck Institute for Biophysical Chemistry, Department of Molecular Biology, Am Fassberg 11, Göttingen, 37077 Germany; 2 Department of Cellular Biochemistry, University Medical Center Göttingen, Humboldtallee 23, Göttingen, 37073 Germany; 3 Universität Würzburg, Lehrstuhl für Organische Chemie I, Am Hubland, Würzburg, 97074 Germany

**Keywords:** Biochemistry, RNA, Cryoelectron microscopy

## Abstract

Remdesivir is the only FDA-approved drug for the treatment of COVID-19 patients. The active form of remdesivir acts as a nucleoside analog and inhibits the RNA-dependent RNA polymerase (RdRp) of coronaviruses including SARS-CoV-2. Remdesivir is incorporated by the RdRp into the growing RNA product and allows for addition of three more nucleotides before RNA synthesis stalls. Here we use synthetic RNA chemistry, biochemistry and cryo-electron microscopy to establish the molecular mechanism of remdesivir-induced RdRp stalling. We show that addition of the fourth nucleotide following remdesivir incorporation into the RNA product is impaired by a barrier to further RNA translocation. This translocation barrier causes retention of the RNA 3ʹ-nucleotide in the substrate-binding site of the RdRp and interferes with entry of the next nucleoside triphosphate, thereby stalling RdRp. In the structure of the remdesivir-stalled state, the 3ʹ-nucleotide of the RNA product is matched and located with the template base in the active center, and this may impair proofreading by the viral 3ʹ-exonuclease. These mechanistic insights should facilitate the quest for improved antivirals that target coronavirus replication.

## Abstract (web summary)

Remdesivir is a nucleoside analog that inhibits the SARS-CoV-2 RNA dependent RNA polymerase (RdRp) and is used as a drug to treat COVID19 patients. Here, the authors provide insights into the mechanism of remdesivir-induced RdRp stalling by determining the cryo-EM structures of SARS-CoV-2 RdRp with bound RNA molecules that contain remdesivir at defined positions and observe that addition of the fourth nucleotide following remdesivir incorporation into the RNA product is impaired by a barrier to further RNA translocation.

## Introduction

Coronaviruses use an RdRp enzyme to carry out replication and transcription of their RNA genome^1–5^. The RdRp consists of three non-structural protein (nsp) subunits, the catalytic subunit nsp12^6^ and the accessory subunits nsp8 and nsp7^3,7^. Structures of the RdRp of SARS-CoV-2 were obtained in free form^8^ and with RNA template-product duplexes^9–11^. Together with a prior structure of SARS-CoV RdRp^12^, these results have elucidated the RdRp mechanism. For RNA-dependent RNA elongation, the 3ʹ-terminal nucleotide of the RNA product chain resides in the –1 site and the incoming nucleoside triphosphate (NTP) substrate binds to the adjacent +1 site. Catalytic nucleotide incorporation then triggers RNA translocation and liberates the +1 site for binding of the next incoming nucleoside triphosphate (NTP).

The nucleoside analog remdesivir is the only FDA-approved drug for the treatment of COVID-19 patients^13–16^. Remdesivir inhibits the RdRp of coronaviruses^10,17–22^ and shows antiviral activity in cell culture and animals^21,23–25^. Remdesivir is a phosphoramidate prodrug that is metabolized in cells to yield an active NTP analog^21^ that we refer to as remdesivir triphosphate (RTP). Biochemical studies showed that the RdRp can use RTP as a substrate, leading to the incorporation of remdesivir monophosphate (RMP) into the growing RNA product^10,20,22^. After RMP incorporation, the RdRp extends RNA by three more nucleotides before it stalls^10,20,22^. This stalling mechanism is specific to coronaviruses because the RdRp of Ebola virus can add five RNA nucleotides after RMP incorporation before it stalls^26^.

Recent structural studies showed RdRp-RNA complexes after remdesivir addition to the RNA product 3ʹ-end. One structure contained RMP in the +1 site^9^, whereas another structure contained RMP in the –1 site^10^. In both structures, RMP mimics adenosine monophosphate (AMP) and forms standard Watson–Crick base pairs with uridine monophosphate (UMP) in the RNA template strand. Thus, these studies explained how RMP is incorporated into RNA instead of AMP. However, they do not explain how remdesivir inhibits the RdRp because RdRp stalling occurs only after three more nucleotides have been added to the RNA^10,20^.

## Results

### Biochemical reconstitution of RdRp stalling by remdesivir

**Fig. 1:**
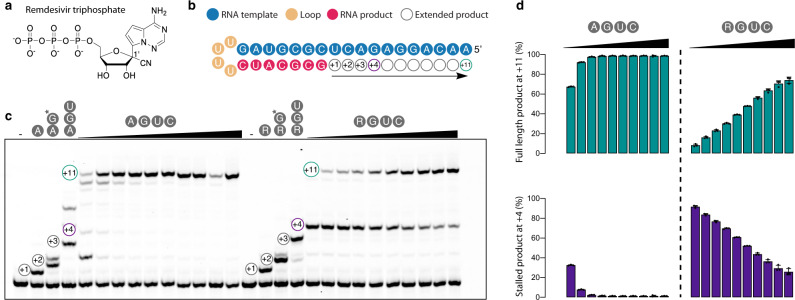
Remdesivir impairs RNA elongation by RdRp. **a** Chemical structure of remdesivir triphosphate (RTP) showing the ribose 1ʹ cyano group. **b** RNA template-product duplex. The direction of RNA elongation is indicated. **c** Remdesivir-induced RdRp stalling. Replacing ATP with RTP leads to an elongation barrier after addition of three more nucleotides. The barrier can be overcome at higher NTP concentrations. The RNA 5ʹ-end contains a fluorescent label. Asterisk indicates 3ʹ-dGTP. Source data are provided as a Source Data file. **d** Quantification of the experiment in panel **c** after triplicate measurements. Standard deviations are shown. Source data are provided as a Source Data file.

To investigate remdesivir-induced RdRp stalling, we first investigated how RTP (Fig. 1a) influences RdRp elongation activity on an RNA template-product scaffold (Fig. 1b) using a highly defined biochemical system (Methods). Consistent with recent studies^20,22^, we observed that RMP is readily incorporated into RNA and that the RNA is subsequently elongated by three more nucleotides before the RdRp stalls (Fig. 1c, d). At high NTP concentrations, RdRp stalling was largely overcome and the full-length RNA product was formed despite the presence of RMP in the RNA product (Fig. 1c, d). Thus, the predominant mechanism of remdesivir action after its incorporation into the growing RNA is delayed RdRp stalling. Although we cannot exclude that a minor fraction of RdRp-RNA complexes may dissociate and terminate elongation, the stalling mechanism is also observed in a recent single-molecule study^27^.

### Preparation of remdesivir-containing RNA oligonucleotides

**Fig. 2:**
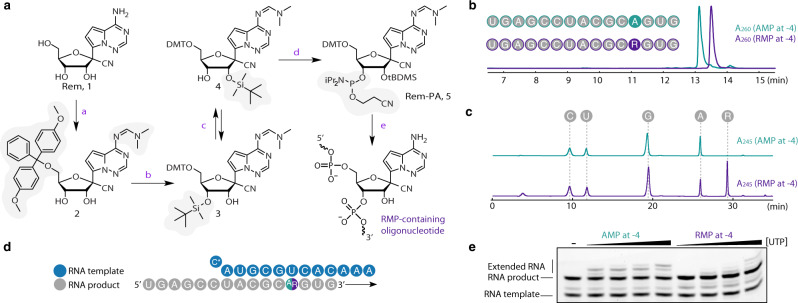
Preparation of remdesivir-containing RNA. **a** Scheme of the synthesis of 5ʹ-*O*-DMT-2ʹ-*O*-TBDMS-protected 3ʹ-cyanoethyl diisopropyl phosphoramidite (Rem-PA), which was used to synthesize RNA oligos with remdesivir monophosphate (RMP) at defined positions. **b** Analysis of RMP-containing RNA by denaturing HPLC confirms the presence of RMP. **c** Analysis of the RMP-containing RNA by LC-MS after digestion into mononucleosides confirms the presence of RMP. **d** Minimal RNA template-product scaffold with RMP (R) or AMP (A) in a synthesized RNA oligonucleotide product strand. **e** The presence of RMP in a synthesized RNA oligonucleotide inhibits RNA extension by RdRp on the minimal RNA scaffold (**d**). Source data are provided as a Source Data file.

To uncover the mechanistic basis of remdesivir-induced RdRp stalling, we aimed to determine structures of RdRp-RNA complexes containing RMP at defined positions in the RNA product strand. We prepared RMP-containing RNA oligonucleotides by solid-phase synthesis using 5ʹ-*O*-DMT-2ʹ-*O*-TBDMS-protected 3ʹ-cyanoethyl diisopropyl phosphoramidite (Rem-PA), which we synthesized from 1ʹ-cyano-4-aza-7,9-dideazaadenosine (Rem) in four steps (Fig. 2a, Methods, Supplementary Methods). The presence of RMP in the obtained RNA oligonucleotides was confirmed by denaturing HPLC and LC-MS after digestion into mononucleosides (Fig. 2b, c). We further confirmed that the presence of RMP inhibits RNA extension by RdRp on minimal RNA template-product scaffolds (Fig. 2d, e).

### Structural analysis of RdRp stalling by remdesivir

The ability to prepare RNAs containing RMP at defined positions enabled us to structurally capture the two states of the RdRp complex that are relevant for understanding remdesivir-induced RdRp stalling. Specifically, we investigated RdRp-RNA complexes captured after addition of two or three nucleotides following RMP incorporation. We prepared RNA scaffolds containing RMP at positions –3 or –4 by annealing short RMP-containing oligonuclotides to long, loop-forming RNAs (scaffolds 1 and 2, respectively) (Methods). The annealed RNA scaffolds were then bound to purified RdRp and subjected to cryo-EM analysis as described^11^, resulting in two refined structures (Supplementary Table 1).

**Fig. 3:**
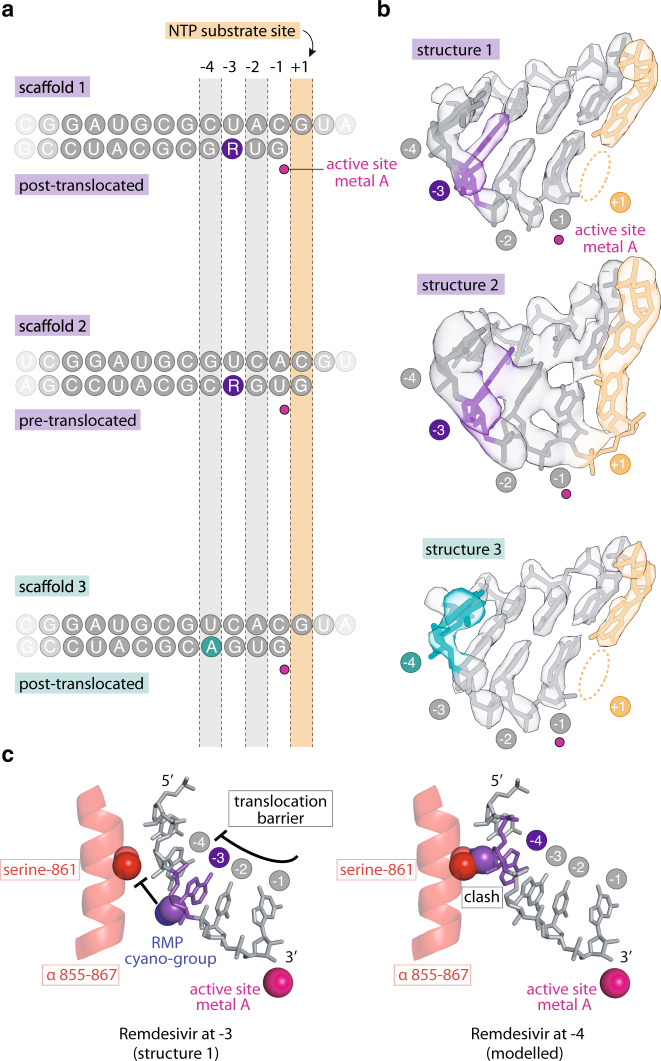
Structural analysis of remdesivir-induced RdRp stalling. **a** Position of RNA scaffolds 1–3 as observed in RdRp-RNA complex structures 1–3. Template and product strands are on the top and bottom, respectively. **b** Cryo-EM density of RNA in the active center of structures 1–3. The active site metal ion was modeled^41^ and is shown as a magenta sphere. **c** The C1ʹ-cyano group of the RMP ribose moiety (violet) is accommodated at position –3 (left), but would clash with the side chain of nsp12 residue serine-861 (red) at position –4 (right). Spheres indicate atomic van der Waals surfaces.

The first RdRp-RNA structure (structure 1) was resolved at 3.1 Å resolution (Supplementary Fig. 1) and showed that the RMP was located at position –3 of the RNA product strand, as expected from the design of scaffold 1 (Fig. 3a, b). The RdRp-RNA complex adopted the post-translocated state. The RNA 3ʹ-end resided in the –1 site and the +1 site was free to bind the next NTP substrate. Comparison with our previous RdRp-RNA complex structure^11^ did not reveal significant differences. The 1ʹ-cyano group of the RMP ribose moiety was located at position –3 and is accommodated there by an open space in the RNA product-binding site of the RdRp (Fig. 3a, b). Thus, structure 1 represents an active state of the elongation complex that is poised to add one more nucleotide to the RNA before stalling, consistent with biochemical results.

### A translocation barrier underlies remdesivir-induced RdRp stalling

The second RdRp-RNA structure (structure 2) was resolved at 3.4 Å resolution (Supplementary Fig. 1) and showed that the RMP moiety was not located at position –4, as was expected from our design of scaffold 2, but was instead located at position –3 (Fig. 3a, b). The +1 site was no longer free, as observed in structure 1, but was occupied by the nucleotide at the RNA 3ʹ-end. The RdRp-RNA complex adopts the pre-translocated state and cannot bind the next NTP substrate. Thus, structure 2 indicates that the RMP moiety in the RNA product strand is not tolerated at position –4. These results suggested that remdesivir-induced stalling of the RdRp is due to impaired translocation of the RNA after the RMP reaches register –3.

To test the hypothesis that RdRp stalling results from a translocation barrier, we formed a third RdRp-RNA complex with an RNA scaffold that was identical to that in structure 2 except that RMP was replaced by AMP, and we determined the resulting structure 3 at 2.8 Å resolution (Fig. 3a, b and Supplementary Fig. 1). In structure 3, the RdRp-RNA complex adopted the post-translocation state and the +1 site was again free, as observed in structure 1. This shows that the unexpected pre-translocated state that we observed in structure 2 was indeed caused by the presence of RMP, which was not tolerated at position –4. In conclusion, the RMP moiety in the RNA product strand gives rise to a translocation barrier that impairs movement of the RMP from position –3 to position –4.

## Discussion

Our results define the structural mechanism of remdesivir-induced RdRp stalling. They show directly that stalling is caused by a translocation barrier that the RdRp encounters after the addition of three more nucleotides following remdesivir incorporation into the growing RNA. Prior observations suggest that the translocation barrier that we define here is caused by the presence of the C1ʹ-cyano group in the remdesivir ribose moiety. First, this cyano group is critical for antiviral potency against Ebola virus^21^. Second, modeling the RMP at position –4 of the RNA product strand results in a steric clash with the side chain of serine-861 in nsp12^10,20^. Indeed, our structural data strongly support the modeling (Fig. 3c). Third, truncation of serine-861 to alanine^10,28^ or glycine^28^ renders the RdRp less sensitive or insensitive, respectively, to inhibition by remdesivir. We conclude that the translocation barrier results from the sterically impaired passage of the cyano group in RMP past the serine-861 side chain in the nsp12 subunit of RdRp.

We have summarized the mechanism of remdesivir-induced RdRp stalling in a molecular animation (Supplementary Movie 1). The remdesivir-stalled state is observed in our structure 2. In this structure, the RNA product 3ʹ-nucleotide is buried in the active center and is base-paired with the RNA template strand (Fig. 3a, b). This may explain why the RNA 3ʹ-end may at least partially escape proofreading by the viral exonuclease nsp14^29,30^. Nevertheless, some proofreading can occur and this renders remdesivir less efficient^19^, indicating that the viral exonuclease can remove several nucleotides from the base-paired RNA 3ʹ-end. Such removal of several RNA nucleotides may require RNA backtracking along the RdRp, and this may be induced by the viral helicase nsp13^31^.

Finally, although delayed RdRp stalling after remdesivir incorporation into growing RNA is likely to be an important mechanism of inhibition, another mechanism of remdesivir action based on RNA template-dependent inhibition of the RdRp was recently proposed^28^. This alternative mechanism and the mechanisms of RdRp-dependent RNA proofreading will be studied further in the future. Meanwhile, the mechanistic insights presented here may facilitate the search for compounds with improved potential to interfere with coronavirus replication.

## Methods

No statistical methods were used to predetermine sample size. The experiments were not randomized, and the investigators were not blinded to allocation during experiments and outcome assessment.

### RNA extension assays

Preparation of SARS-CoV-2 RdRp was carried out as described^11^. Synthetic gene for nsp12 was amplified by PCR (forward primer: 5′-TAC TTC CAA TCC AAT GCA TCT GCT GAC GCT CAG TCC TTC CTG-3′, reverse primer: 5′-TTA TCC ACT TCC AAT GTT ATT ATT GCA GCA CGG TGT GAG GGG-3′) and cloned into pFastBac vector 438C (Addgene #154759). Protein was expressed in Hi5 cells for 60 h. After harvesting by centrifugation, cells were lysed in lysis buffer (300 mM NaCl, 50 mM Na-HEPES pH 7.4, 10% (v/v) glycerol, 30 mM imidazole pH 8.0, 5 mM β-mercaptoethanol, 0.284 μg/ml leupeptin, 1.37 μg/ml pepstatin, 0.17 mg/ml PMSF, 0.33 mg/ml benzamidine and 3 mM MgCl_2_) and opened by sonication. Lysate was clarified and passed through a HisTrap HP 5 ml (GE Healthcare) preequilibrated in lysis buffer, and the protein was eluted with nickel elution buffer (300 mM NaCl, 50 mM Na-HEPES pH 7.4, 10% (v/v) glycerol, 500 mM imidazole pH 8.0, 3 mM MgCl_2_ and 5 mM β-mercaptoethanol) directly onto an XK column 16/20 (GE Healthcare) containing amylose resin. Protein was eluted in amylose elution buffer (300 mM NaCl, 50 mM Na-HEPES pH 7.4, 10% (v/v) glycerol, 116.9 mM maltose, 30 mM imidazole pH 8.0 and 5 mM β-mercaptoethanol). Pure protein fractions were digested with TEV protease overnight. Protein solution was after digestion passed through a HisTrap HP 5 ml column and HiTrap Heparin 5 ml column (GE Healthcare). The flow-through was concentrated and further purified via HiLoad S200 16/600 pg column equilibrated in size exclusion buffer (300 mM NaCl, 20 mM Na-HEPES pH 7.4, 10% (v/v) glycerol, 1 mM MgCl_2_, 1 mM TCEP). Peak fractions were pooled, concentrated until 102 μM, aliquoted, flash-frozen and stored at −80 °C until use.

Synthetic genes for nsp8 and nsp7 were amplified by PCR (nsp8: forward primer: 5′-TAC TTC CAA TCC AAT GCA GCA ATT GCA AGC GAA TTT AGC AGC CTG-3′, reverse primer: 5′-TTA TCC ACT TCC AAT GTT ATT ACT G C AGT TTA ACT GCG CTA TTT GCA CG-3′; nsp7: forward primer: 5′-TAC TTC CAA TCC AAT GCA AGC AAA ATG TCC GAT GTT AAA TGC ACC AGC-3′, reverse primer: 5′-TTA TCC ACT TCC AAT GTT ATT ACT GCA GGG TTG CAC GAT TAT CCA GC-3′) and separately cloned into pET-derived 14-B vector (Addgene #154757 for nsp7 and #154758 for nsp8). Both nsp7 and nsp8 were expressed and purified in the same way. Proteins were overexpressed in E. coli BL21 (DE3) RIL cells. Bacterial cells were induced with 0.5 mM isopropyl β-D-1-thiogalactopyranoside and shaken for 16 h at 18 °C. After expression, cells were centrifuged and immediately lysed by sonication in lysis buffer (300 mM NaCl, 50 mM Na-HEPES pH 7.4, 10% (v/v) glycerol, 30 mM imidazole pH 8.0, 5 mM β-mercaptoethanol, 0.284 μg/ml leupeptin, 1.37 μg/ml pepstatin, 0.17 mg/ml PMSF and 0.33 mg/ml benzamidine). Clarified lysate was loaded onto a HisTrap HP 5 ml (GE Healthcare) preequilibrated in lysis buffer, and the protein was eluted in nickel elution buffer (150 mM NaCl, 50 mM Na-HEPES pH 7.4, 10% (v/v) glycerol, 500 mM imidazole pH 8.0 and 5 mM β-mercaptoethanol). Eluted protein was dialyzed against a dialysis buffer (150 mM NaCl, 50 mM Na-HEPES pH 7.4, 10% (v/v) glycerol and 5 mM β-mercaptoethanol) and simultaneously digested with TEV protease overnight. After digestion, protein was passed through a HisTrap HP 5 ml column and a HiTrap Q 5 ml column (GE Healthcare). Flow-through containing the protein of interest was concentrated and further purified on a HiLoad S200 16/600 pg column equilibrated in size exclusion buffer (150 mM NaCl, 20 mM Na-HEPES pH 7.4, 5% (v/v) glycerol, 1 mM TCEP). Peak fractions were pooled, concentrated, aliquoted, flash-frozen and stored at −80 °C until use. All protein purification steps were done at 4 °C.

RTP synthesis is described in Supplementary Methods. All unmodified RNA oligonucleotides were purchased from Integrated DNA Technologies. The RNA sequence used for the RNA extension assay (Fig. 1c) is /56-FAM/rArArC rArGrG rArGrA rCrUrC rGrCrG rUrArG rUrUrUrU rCrUrA rCrGrC rG. The assay was performed as described^11^, except for the following changes. The final concentrations of nsp12, nsp8, nsp7, and RNA were 3 μM, 9 μM, 9 μM, and 2 μM, respectively. The highest concentration of NTPs was 0.5 mM for each nucleotide (ATP or RTP, GTP, UTP, and CTP), followed by a two-fold serial dilution. RNA products were resolved on a denaturing sequencing gel and visualized by Typhoon 95000 FLA Imager (GE Healthcare Life Sciences). The RNA sequences used for extending RMP-containing RNA oligonucleotides are: rUrGrA rGrCrC rUrArC rGrC- rA or rR- rGrUrG (product) and rArArA rCrArC rUrGrC rGrUrA/3ddC/(template). The extension assay (Fig. 2e) was performed as described^11^, with minor changes. Reactions were started by addition of UTP (final concentrations: 6.25 μM, 12.5 μM, 25 μM, or 250 μM). RNA products were visualized by SYBR Gold (Thermo Fischer) staining and imaged with Typhoon 9500 FLA Imager (GE Healthcare Life Sciences).

### Preparation of RMP-containing RNA oligonucleotides

1ʹ-cyano-4-aza-7,9-dideazaadenosine (Rem, **1**) was converted to the 5ʹ-*O*-DMT-2ʹ-*O*-TBDMS-protected 3ʹ-cyanoethyl diisopropyl phosphoramidite (Rem-PA, **5**) in four steps. Details of the synthetic procedures and nuclear magnetic resonance spectra of isolated compounds are given in the Supplementary Methods. RNA oligonucleotides were then prepared by solid-phase synthesis on CPG support (0.6 µmol scale) using 2ʹ-*O*-TOM-protected ribonucleoside phosphoramidites (100 mM in CH_3_CN) and ethylthiotetrazol (ETT, 250 mM in CH_3_CN) as activator, with 4 min coupling time. Rem-PA was used as freshly prepared solution (100 mM) in dry 1,2-dichloroethane, with a coupling time of two times 12 min. Detritylation was performed with 3% dichloroacetic acid in dichloromethane. Capping solutions contained 4-dimethylamino pyridine (0.5 M) in acetonitrile for Cap A, and acetic anhydride/sym-collidine/acetonitrile (2/3/5) for Cap B. Oxidation was performed with iodine (10 mM) in acetonitrile/sym-collidin/water (10/1/5). The oligonucleotides were deprotected with 25% NH_4_OH at 25 °C for 30 h, followed by 1 M TBAF in THF for 12 h, and purified by denaturing polyacrylamide gel electrophoresis.

### Analysis of RMP-containing RNA oligonucleotides

The purity and identity of the RNA oligonucleotides was analyzed by anion-exchange HPLC (Dionex DNAPac PA200, 2 × 250 mm, at 60 °C. Solvent A: 25 mM Tris-HCl (pH 8.0), 6 M Urea. Solvent B: 25 mM Tris-HCl (pH 8.0), 6 M Urea, 0.5 M NaClO_4_. Gradient: linear, 0–40% solvent B, 4% solvent B per 1 CV), and HR-ESI-MS (Bruker micrOTOF-Q III, negative ion mode, direct injection). An aliquot (200 pmol in 25 µL) was digested by snake venom phosphodiesterase (SVPD, 0.5 U) in the presence of bacterial alkaline phosphatase (BAP, 0.5 U) in 40 mM Tris. pH 7.5, 20 mM MgCl_2_, and the resulting mononucleosides were analyzed by liquid chromatography–electrospray ionization–tandem mass spectrometry using an RP-18 column (Synergi 4 µm Fusion-RP C18 80 Å, 250 × 2 mm, at 25 °C. aqueous mobile phase A: 5 mM NH_4_OAc, pH 5.3. organic mobile phase B: 100% acetonitrile. Gradient: 0–5% B in 15 min, then 5–50% B in 20 min, flow rate 0.2 ml/min) and micrOTOF-Q III with ESI ion source operated in positive ion mode (capillary voltage: 4.5 kV, end plate offset: 500 V, nitrogen nebulizer pressure: 1.4 bar, dry gas flow: 9 l/min). Extracted ion chromatograms and UV absorbance traces at 245 nm confirmed presence of remdesivir.

### Cryo-EM sample preparation and data collection

SARS-CoV-2 RdRp was prepared as described^11^. The RNA scaffolds used for structural studies were comprisesd of two RNA strands. The first RNA strand forms the RNA template-product hairpin and lacks the last 15 nt at the 3ʹ end. The second strand contains the missing 15 nt sequence. Upon scaffold annealing, a complete RNA template-product is formed with a single nick in the product RNA. This strategy had to be used because the length of the remdesivir-containing oligonucleotides was limited due to technical reasons. RNA scaffolds for RdRp-RNA complex formation were prepared by mixing equimolar amounts of two RNA strands in annealing buffer (10 mM Na-HEPES pH 7.4, 50 mM NaCl) and heating to 75 °C, followed by step-wise cooling to 4 °C. RNA sequences for RMP at position -3 (structure 1) are: rUrUrU rUrCrA rUrGrC rArUrC rGrCrG rUrArG rGrCrU rCrArU rArCrC rGrUrA rUrUrG rArGrA rCrCrU rUrUrU rGrGrU rCrUrC rArArU rArCrG rGrUrA and rUrGrA rGrCrC rUrArC rGrCrG rRrUrG. RNA sequences for AMP/RMP at position -4 (structures 2 and 3) are: rUrUrU rUrCrA rUrGrC rArCrU rGrCrG rUrArG rGrCrU rCrArU rArCrC rGrUrA rUrUrG rArGrA rCrCrU rUrUrU rGrGrU rCrUrC rArArU rArCrG rGrUrA and rUrGrA rGrCrC rUrArC rGrC- rA/rR -rGrUrG. RdRp-RNA complexes were formed by mixing purified nsp12 (scaffold 1:2 nmol, scaffold 2:2 nmol, scaffold 3:1.6 nmol) with an equimolar amount of annealed RNA scaffold and threefold molar excess of each nsp8 and nsp7. After 10 min of incubation at room temperature, the mixture was applied to a Superdex 200 Increase 3.2/300 size exclusion chromatography column, which was equilibrated in complex buffer (20 mM Na-HEPES pH 7.4, 100 mM NaCl, 1 mM MgCl_2_, 1 mM TCEP) at 4 °C. Peak fractions corresponding to the RdRp-RNA complex were pooled and diluted to ~2 mg/ml. For structure 2, an additional 0.36 nmol of annealed RNA scaffold were spiked into the sample prior to grid preparation. Three microliters of the concentrated RdRp-RNA complex were mixed with 0.5 µl of octyl ß-d-glucopyranoside (0.003% final concentration) and applied to freshly glow discharged R 2/1 holey carbon grids (Quantifoil). The grids were blotted for 5 s using a Vitrobot MarkIV (Thermo Fischer Scientific) at 4 °C and 100% humidity and plunge frozen in liquid ethane.

Cryo-EM data were collected using SerialEM^32^ on a 300 keV Titan Krios transmission electron microscope (Thermo Fischer Scientific). Prior to detection, inelastically scattered electrons were filtered out with a GIF Quantum energy filter (Gatan) using a slit width of 20 eV. Images were acquired in counting mode (non-super resolution) on a K3 direct electron detector (Gatan) at a nominal magnification of 105,000x resulting in a calibrated pixel size of 0.834 Å/pixel. Images were exposed for a total of 2.2 s with a dose rate of 19 e^−^/px/s resulting in a total dose of 60 e^−^/Å^2^, which was fractionated into 80 frames. Previous cryo-EM analysis of the SARS-CoV2 RdRp showed strong preferred orientation of the RdRp particles in ice^11^. Therefore, all data were collected with 30° tilt to obtain more particle orientations. Motion correction, contrast transfer function (CTF)-estimation, and particle picking and extraction were performed on the fly using Warp^33^. In total, 8004, 11,764, and 7043 movies were collected for structures 1, 2, and 3, respectively.

### Cryo-EM data processing and analysis

For structure 1, 1.8 million particles were exported from Warp^33^ 1.0.9 to cryoSPARC^34^ 2.15. After ab initio refinement of five classes, an intermediate map from the previous processing of EMD-11007^11^ was added as a 6th reference, and supervised three-dimensional (3D) classification was performed. 654k particles (37%) from the best class deemed to represent the polymerase were subjected to 3D refinement to obtain a 3.1 Å map. Half-maps and particle alignments were exported to M^35^ 1.0.9, where reference-based frame series alignment with a 2 × 2 image-warp grid, as well as CTF refinement were performed for two iterations. Although the resulting map had the same 3.1 Å nominal resolution, the features of interest were significantly cleaner.

For structure 2, 3.4 million particles were exported from Warp 1.0.9 to cryoSPARC 2.15. After ab initio refinement of 5 classes, the EMD-11007-related reference was added as a 6th reference, and supervised 3D classification was performed. To further clean up the resulting 881k particles (26%) from the best class deemed to represent the polymerase, another ab initio refinement of five classes was performed. Three of these classes and the EMD-11007-related reference were used for supervised 3D classification. 474k particles (54%) from the best class deemed to represent the polymerase were subjected to 3D refinement to obtain a 3.6 Å map. Half-maps and particle alignments were exported to M 1.0.9, where reference-based frame series alignment with a 2 × 2 image-warp grid, as well as CTF refinement were performed for two iterations to obtain a 3.4 Å map.

For structure 3, 2.2 million particles were exported from Warp 1.0.9 to cryoSPARC 2.15. Initial unsupervised 3D classification in three classes was performed using the EMD-11007-related reference. To further clean up the resulting 1.1 million particles (23%), ab initio refinement of five classes was performed. Four of these classes and the EMD-11007-related reference were used for supervised 3D classification. 819k particles (70%) from the best class deemed to represent the polymerase were subjected to 3D refinement to obtain a 3.1 Å map. Half-maps and particle alignments were exported to M 1.0.9, where reference-based frame series alignment with a 2 × 2 image-warp grid, as well as CTF refinement were performed for three iterations to obtain a 2.8 Å map.

### Model building and refinement

Models were built using our previously published SARS-CoV-2 RdRp structure as starting model (PDB 6YYT [10.2210/pdb6YYT/pdb])^11^. For each of the structures 1–3, the model was first rigid-body fit into the density and subsequently adjusted in real-space in Coot^36^. Parts of the N-terminal NiRAN domain of nsp12, the N-terminal extension of nsp8a and the entire nsp8b molecule were removed due to absence or poor quality of density for these regions. Restraints for RMP were generated in phenix.elbow^37^ and the structures were refined using phenix.real_space_refine^38^ with appropriate secondary structure restraints. Model quality was assessed using MolProbity within Phenix^39^, which revealed excellent stereochemistry for all three structural models (Supplementary Table 1). Figures were prepared with PyMol and ChimeraX^40^.

### Reporting summary

Further information on experimental design is available in the Nature Research Reporting Summary linked to this paper.

## Supplementary information

Supplementary Information

Peer Review File

Description of Additional Supplementary Files

Supplementary Movie 1

Reporting Summary

## Data Availability

The electron density reconstructions and structure coordinates were deposited with the Electron Microscopy Database (EMDB) under accession codes EMD-11993, EMD-11994, and EMD-11995 and with the Protein Data Bank (PDB) under accession codes 7B3B, 7B3C, and 7B3D. Other data are available from corresponding authors upon reasonable request. Source data are provided with this paper.

## Source data

Source Data
