# Supplementary material for: Mechanism of SARS-CoV-2 polymerase stalling by remdesivir
Source: Nat Commun. 2021 Jan 12;12:279. doi: 10.1038/s41467-020-20542-0 (PMC7804290; doi:10.1038/s41467-020-20542-0)
Supplement: Supplementary file 1 — Supplementary Information [file 41467_2020_20542_MOESM1_ESM.pdf]

Mechanism of SARS-CoV-2 polymerase stalling by remdesivir

Goran Kokic[1*], Hauke S. Hillen[1,2*], Dimitry Tegunov[1*], Christian Dienemann[1*], Florian Seitz[3*], Jana Schmitzova[1], Lucas Farnung[1], Aaron Siewert[3], Claudia Höbartner[3], and Patrick Cramer[1]

[1]Max Planck Institute for Biophysical Chemistry, Department of Molecular Biology, Am Fassberg 11, 37077 Göttingen, Germany. [2]Current address: University Medical Center, Georg-August-Universität Göttingen. [3]Universität Würzburg, Lehrstuhl für Organische Chemie I, Am Hubland, 97074 Würzburg, Germany. [*]These authors contributed equally. Correspondence: claudia.hoebartner@uni-wuerzburg.de, patrick.cramer@mpibpc.mpg.de

Table of Contents

Supplementary Figure 1 2

Supplementary Table 1 3-4

Supplementary methods 5

Experimental procedures for synthesis of RTP (to Figure 1) 5-8

Experimental procedures for synthesis of Rem-PA (to Figure 2) 9-14

NMR Spectra 15-23

Supplementary References 24

Supplementary Figures related to Supplementary methods

Supplementary Figure 2. Synthesis of RTP 5

Supplementary Figure 3. AIEX chromatograms and UV spectra of RTP and RMP 8

Supplementary Figure 4. Synthesis of Rem-PA 9

Supplementary Table related to Supplementary methods

Supplementary Table 2. Sequences and HR-ESI-MS of synthetic oligonucleotides 9

Supplementary Figure 1

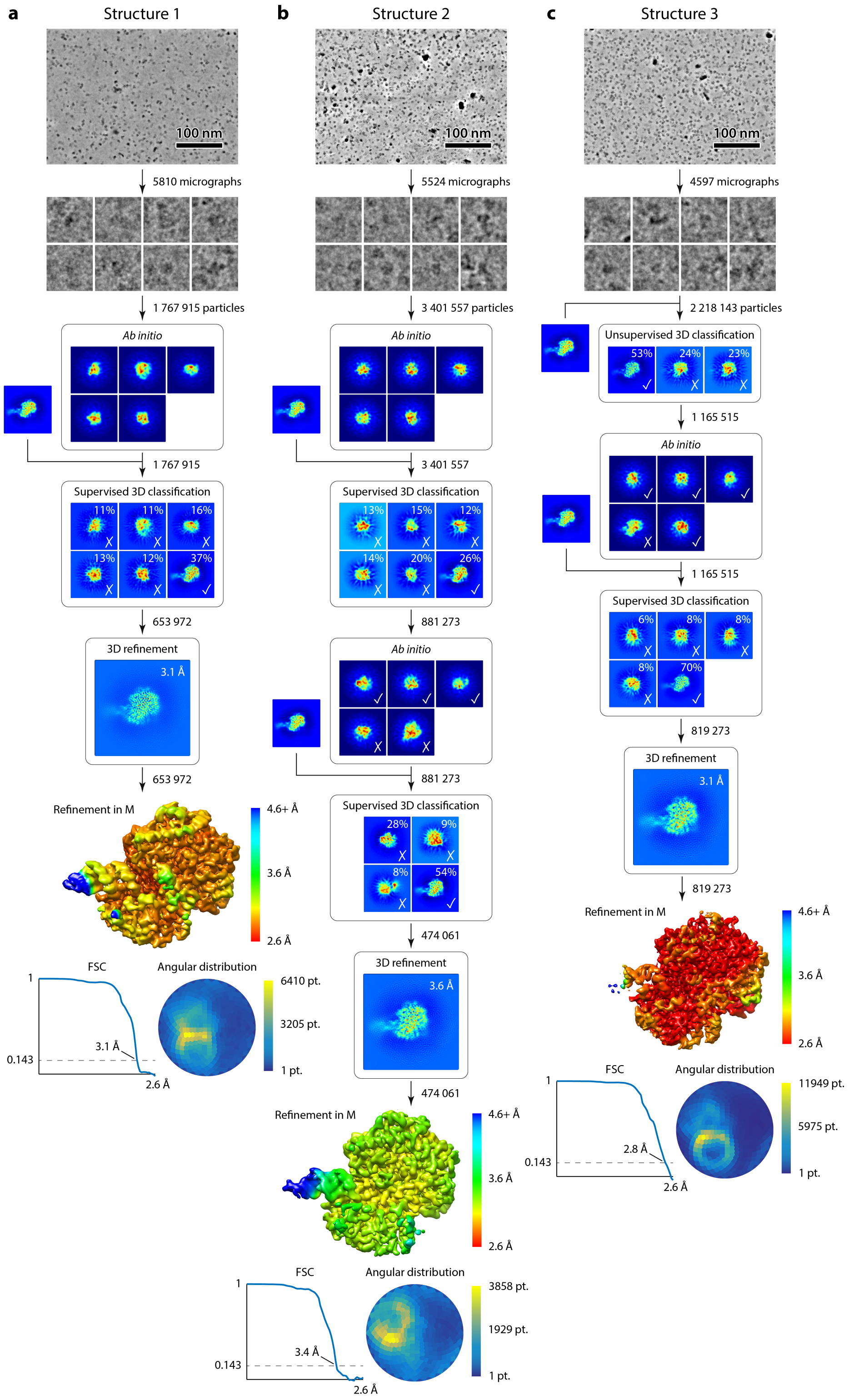

Supplementary Figure 1 | Cryo-EM sorting trees and quality of reconstructions. Related to Figure 3. Cryo-EM sorting tree (top); local resolution, FSC plot and angular distribution of the final reconstruction (bottom) for RMP-containing RdRp-RNA structure 1 **(a)**, RMP-containing RdRp-RNA structure 2 **(b)**, and RdRp-RNA structure 3 **(c)**.

Supplementary Table 1

Supplementary Table 1 | Cryo-EM data collection, refinement, and validation statistics

	**SARS-CoV-2 RdRp structure 1 (PDB 7B3B)**	**SARS-CoV-2 RdRp structure 2 (PDB 7B3C)**	**SARS-CoV-2 RdRp structure 3 (PDB 7B3D)**
	Map 1 (EMD-**11993**)	Map 2 (EMD- 11994)	Map 3 (EMD- 11995)
**Data collection and processing**			
Magnification		105,000 x	
Voltage (kV)		300	
Electron exposure (e–/Å^2)		60	
Defocus range (μm)	0.4—1.7	0.4—2.4	0.5—2.1
Pixel size (Å)		0.834	
Symmetry imposed		C1	
Initial particle images (no.)	1,767,915	3,401,557	2,218,143
Processing pixel size (Å)	0.834 / 1.2	0.834 / 1.3	0.834 / 1.3
Final particle images (no.)	653,972	474,061	819,273
Map resolution (Å)	3.1	3.4	2.8
FSC threshold	0.143	0.143	0.143
Map resolution range (Å)	2.6—3.8	3.0—5.6	2.6—4.1
Map sharpening *B* factor (Å^2)	-110	-122	-96
**Refinement**			
Initial model used (PDB code)	6YYT	6YYT	6YYT
Model resolution (Å)	3.0	3.4	2.8
FSC threshold	0.5	0.5	0.5
Model resolution range (Å)	2.6 - 3.8	3.0 – 5.6	2.6 – 4-1

Model resolution range (Å)	2.6 - 3.8	3.0 – 5.6	2.6 – 4-1
Model composition	8407	8430	8405
Non-hydrogen atoms	991	991	991
Protein residues	22	23	22
Nucleic acids<br>Ligands	2	2	2
B factors (Å^2)			
Protein	57.73	65.61	42.28
Nucleotide	80.10	91.89	59.26
Ligand	50.54	71.81	42.47
R.m.s. deviations			
Bond lengths (Å)	0.003	0.003	0.003
Bond angles (°)	0.886	0.866	0.856
Validation			
MolProbity score	1.16	1.40	1.25
Clashscore	2.80	4.93	4.75
Poor rotamers (%)	0.57	0.00	0.00
Ramachandran plot			
Favored (%)	97.55	97.24	98.67
Allowed (%)	2.45	2.76	1.33
Disallowed (%)	0.00	0.00	0.00

Supplementary Methods

All reactions were performed under inert nitrogen atmosphere with dry solvents. Reagents used for synthesis were purchased in 'pro analysis' or 'pro synthesis' quality and used without further purification. Solvents used for synthesis were purchased in 'puriss. over molecular sieves', 'pro analysis' or 'pro synthesis' quality and used without further purification. For column chromatography, solvents in technical quality were purchased and purified by distillation. For solid-phase synthesis, acetonitrile and dichloroethane were dried over molecular thieves. Thin layer chromatography (TLC) was performed on aluminum plates pre-coated with silica gel 60 F_{254} (Merck). Substances were detected based on fluorescence quenching at 254 nm. For column chromatography, silica gel 60 (Merck) with a particle size of 40 – 63 µm was used. NMR spectra were recorded using Bruker Avance III (400 MHz) spectrometers. Chemical shifts (*δ*) are given in ppm and were referenced using the deuterated solvent as internal standard. Data are reported as: s = singlet, d = doublet, t = triplet, q = quartet, m = multiplet, br = broad; Coupling constants (*J*) are given in Hz. High-resolution (HR) electrospray ionization (ESI) mass spectra (MS) were recorded on a Bruker micrOTOF-Q III spectrometer. The detected mass-to-charge ratio (*m*/*z*) is given, as well as the calculated monoisotopic mass.

<u>Experimental procedures and compound characterization for RTP (to Figure 1)</u>

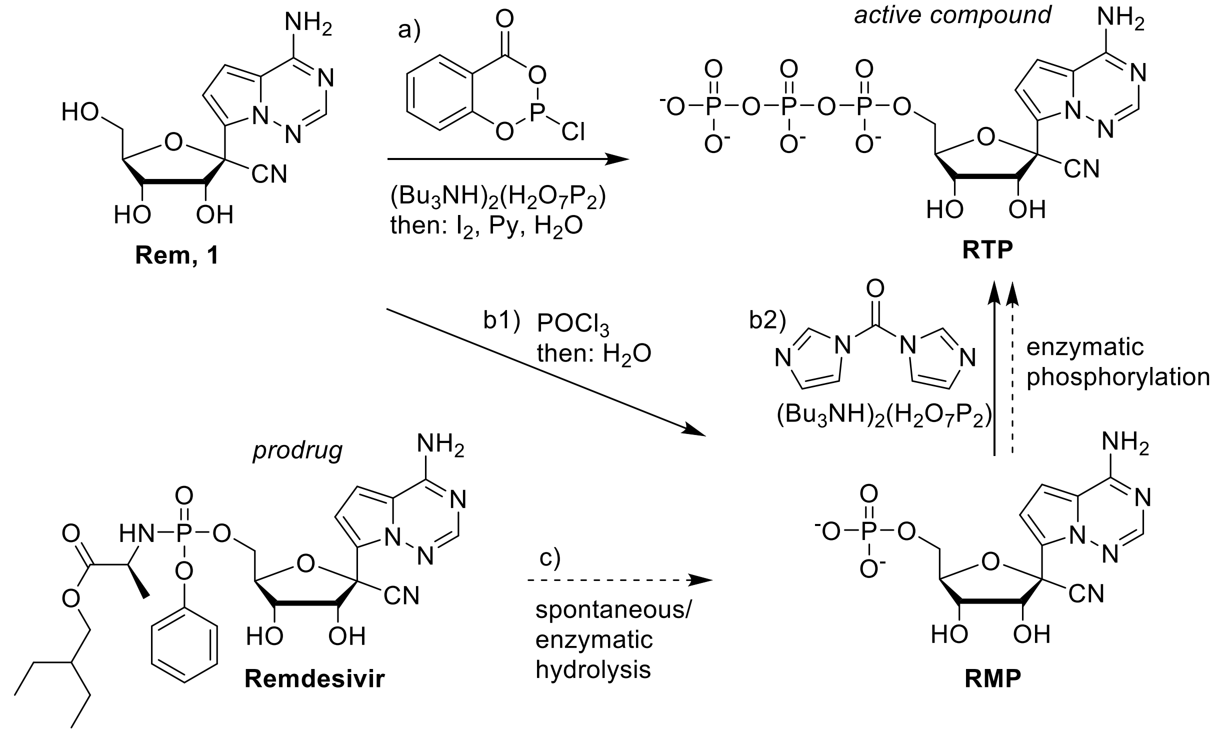

Supplementary Figure 2. Synthesis of RTP. a) one-pot synthesis with salicyl chlorophosphite, Bu_3N, bis(tributylammonium)pyrophosphate in DMF followed by oxidation with iodine in aqueous pyridine. [1] b) two-step synthesis with isolation of RMP. b1) $POCl_3$, $PO(OMe)_3$, then Et_3NHCO_3 buffer. b2) carbonyldiimidazole, bis(tributylammonium) pyrophosphate in DMF. c) enzymatic (intracellular) activation of Remdesivir for comparison.

1'-Cyano-4-aza-7,9-dideazaadenosine 5'-triphosphate (**RTP**) triethylammonium salt

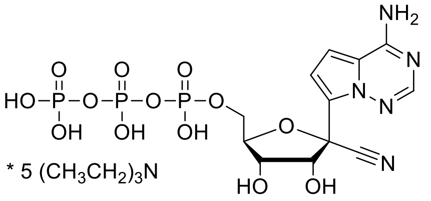

RTP
$C_{12}H_{16}N_5O_{13}P_3$
531.20 g•mol^{-1}

a) one-pot procedure following a general method described by Caton-Williams et al.[1]

Bis(tributylammonium)pyrophosphate (154 mg, 280 µmol, 2.00 eq) was dissolved in dry DMF (0.48 mL), tri-*n*-butylamine (520 µL, 2.2 mmol, 16 eq) was added, and the reaction mixture was stirred at ambient temperature for 5 min. A solution of salicyl chlorophosphite (57 mg, 280 µmol, 2.00 eq) in anhydrous DMF (0.48 mL) was added, and the reaction mixture was stirred vigorously at ambient temperature for 30 min.

Two equivalents of the in-situ generated triphosphorylation reagent were added at 0°C to 1'-cyano-4-aza-7,9-dideazadenosine (**1**, 19.5 mg, 67 µmol, 1.00 eq.). After removal of the ice bath, the reaction mixture was stirred for 3 h at ambient temperature. A solution of iodine (20 mM in pyridine/water 9:1, ca. 1.1 mL) was added stepwise, until a brown color persisted for at least 15 min. Two reaction volumes of ultrapure water (ca. 3.3 mL) were added, followed by stirring for 1.5 h at ambient temperature. An aqueous solution of sodium chloride (20% in water), followed by ethanol (ca. 22 mL) were added, and the reaction mixture kept on dry ice for 1 h. The crude mixture was centrifuged for 10 min at -9°C (3200g), the supernatant removed, and the air dried pellet was purified by RP HPLC using a gradient of 3 % to 10 % acetonitrile in triethylammonium acetate buffer (50 mM, pH 7.5). The purest fractions were pooled, the solvent removed by lyophilization, and the product dissolved in water (350 µL) to yield a stock solution of **RTP.** The concentration was determined by UV spectroscopy on a NanoDrop One spectrometer (Thermo Fisher Scientific) using ε^{245} = 37350 M^{-1}cm^{-1} to give a concentration of 10 mM (3.5 µmol, 5.2 % yield).

^{1}H NMR (400 MHz, D_2O): δ (ppm) = 7.92 (s, 1H, C2-H), 7.08 (d, *J* = 4.8 Hz ,1H, C5-H), 6.94 (d, *J* = 4.7 Hz ,1H, C6-H), 5.02 (d, *J* = 5.3 Hz, 1H, C2'-H), 4.60 (dd, *J* = 5.3 and 3.1 Hz, 1H, C4'-H), 4.51 (m, 1H, C3'-H), 4.21 (m, 1H, C5'-H), 4.01 (ddd, 1H, *J* = 11.8, 4.7 and 3.1 Hz, C5'-H), 3.15 (q, ~30H, CH_2N in triethylamine), 1.23 (t, ~45H, CH_3 in triethylamine).

^{13}C (gHSQCAD) NMR (101 MHz, D_2O): δ (ppm) = 147.30 (C2), 111.3 (C5), 102.4(C6), 85.5 (C3'), 74.9 (C2'), 70.1 (C4'), 64.7 (C5'), resonances of the protonated carbon atoms were recorded.

^{31}P{^{1}H} NMR (394 MHz, D_2O): δ (ppm) = -6.5 (d, *J* = 20 Hz), -11. 4 (d, *J* = 20 Hz),-22.5 (t, *J* = 20 Hz).

HR-ESI-MS: *m/z* calc. ($C_{12}H_{15}N_5O_{13}P_3$ [M-H]$^-$): 529.98792, found: 529.98842.

Analytical data are consistent with previously reported values.[2]

b) Two-step procedure with isolation of RMP

1'-Cyano-4-aza-7,9-dideazaadenosine 5'-monophosphate (**RMP**)

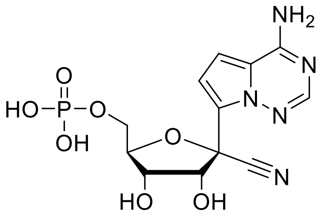

RMP
$C_{12}H_{14}N_5O_7P$
371.25 g•mol^{-1}

1'-cyano-4-aza-7,9-dideazadenosine (**1**, 51 mg, 0.17 mmol, 1.00 eq.) was dissolved in dry trimethylphosphate (2.5 mL), cooled to 0 °C, treated with $POCl_3$ (0.3 mL, 0.34 mmol, 1.90 eq.) and stirred at 0 °C for 4 h. A solution of tributylamine (0.2 mL, 0.84 mmol, 4.9 eq.) and bis(tributylammonium)pyrophosphate (150 mg, 0.27 mmol, 1.6 eq.) in dry MeCN (5 mL) was added and the mixture stirred at 0 °C for 0.5 h.) Triethylammonium bicarbonate (TEAB) buffer (1 M, pH 7.5, 2 mL) was added at 0 °C and the mixture stirred at ambient temperature for 0.5 h. The solvent was removed under reduced pressure and coevaporated with water. The crude product was subjected to ion exchange chromatography using a gradient of 0 % to 100 % TEAB buffer (1 M, pH 7.5) in water. After evaporation, the residue was further purified by RP HPLC using a gradient of 0 % to 40 % acetonitrile in triethylammonium acetate buffer (100 mM, pH 7.0). The solvent was removed in high vacuum to yield **RMP** as the triethylammonium salt (37 mg, 0.08 mmol, 46%). In contrast to a previous report,[2] no triphosphate was obtained, most likely because the pyrophosphate solution contained traces of water or the reaction time was too short. The obtained monophosphate was used as a reference compound for analysis of oligonucleotide digestion experiments and determination of the extinction coefficient (see UV spectrum in Figure S2b). The triphosphate (RTP) was obtained upon activation of the monophosphate in the next step.

^{1}H NMR (400 MHz, D_2O): δ (ppm) = 7.78 (s, 1H, C2-H), 6.84 (d, *J* = 4.7 Hz ,1H, C5-H), 6.67 (d, *J* = 4.7 Hz ,1H, C6-H), 4.86 (d, *J* = 5.5 Hz, 1H, C2'-H), 4.46 - 4.44 (m, 1H, C4'-H), 4.39 (dd, *J* = 4.3, 4.0 Hz, 1H, C3'-H), 4.02 - 3.99 (m, 2H, C5'-H).

^{13}C{^{1}H} NMR (125 MHz, D_2O): δ (ppm) = 154.30 (1C, C6), 145.87 (1C, C2), 123.20 (1C, C7), 116.88 (1C, CN), 115.80 (1C, C5), 111.10 (1C, C9), 102.72 (1C, C8), 84.95 (1C, C4'), 76.51 (1C, C1'), 74.68 (1C, C2'), 70.17 (1C, C3'), 63.99 (1C, C5').

^{31}P{^{1}H} NMR (202 MHz, D_2O): δ (ppm) = 0.47 (s).

HR-ESI-MS: *m*/*z* calc. ($C_{12}H_{13}N_5O_7P$ [M-H]$^-$): 370.05581, found: 370.05678.

1'-Cyano-4-aza-7,9-dideazaadenosine 5'-triphosphate (**RTP**)

The **RMP** triethylammonium salt (20 mg, 42 µmol, 1.00 eq.) was dissolved in dry DMF (1 mL), a solution of 1,1'-carbonyldiimidazole (25 mg, 151 µmol, 3.6 eq.) in dry DMF (0.5 mL) was added and the mixture stirred at ambient temperature for 18 h. Methanol (4.9 µL) was added and stirred at ambient temperature for 1 h. A solution of bis(tributylammonium)pyrophosphate (93 mg, 170 µmol, 4.1 eq.) in dry DMF (1 mL) was added and the mixture stirred at ambient temperature for 22 h. The solution was cleared from the precipitate, and the solvent was removed under reduced pressure. The residue was

dissolved in water, washed with dichloromethane, and then water was removed under reduced pressure. The crude product was purified by ion exchange HPLC using a gradient of 0 % to 100 % TEAB buffer (1 M, pH 7.5) in water. The solvent was removed in high vacuum and the product dissolved in water to yield a solution of **RTP.** The concentration was determined by UV spectroscopy on a Cary 100 Bio spectrometer using $\varepsilon^{245} = 37350\ \text{M}^{-1}\text{cm}^{-1}$ to give a yield of (16 mM, 800 µL, 12.7 µmol, 30 %). The spectroscopic data were consistent with the ones reported in a) (see above). In addition, RTP and RMP were analyzed by anion exchange HPLC.

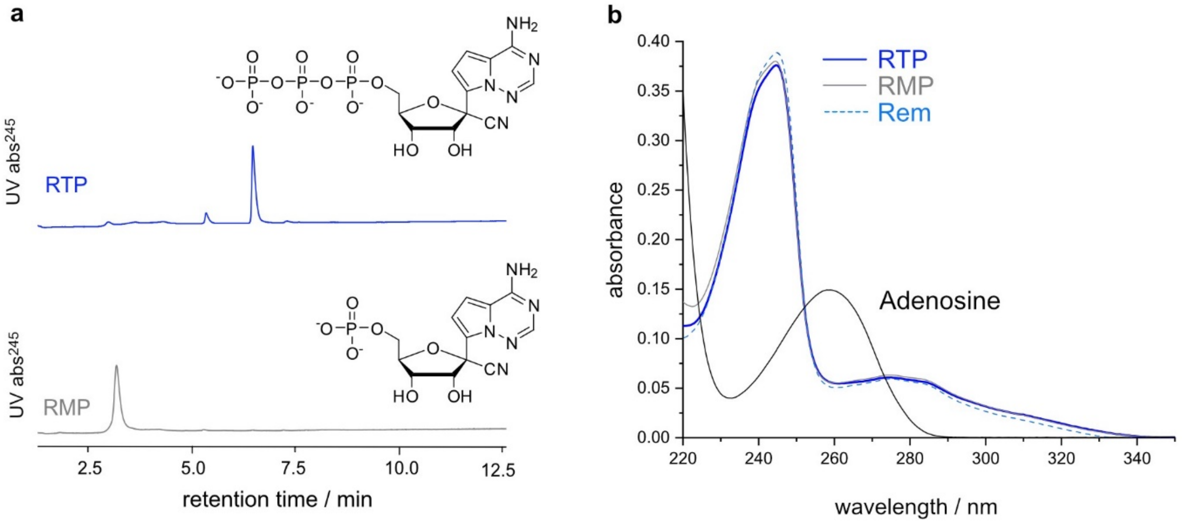

Supplementary Figure 3. Characterization of synthetic RMP and RTP. a) Anion exchange HPLC on Dionex DNA Pac PA200, elution with linear gradient of sodium perchlorate in Tris.HCl buffer (25 mM, pH 8.0); UV Absorbance monitored at 245 nm. b) UV absorbance spectra of solutions of RTP, RMP, and Rem, in comparison to adenosine, in 10 mM Na phosphate buffer pH 7.4. c = 10 µM, d = 1 cm.

Experimental procedures and compound characterization of Rem-PA (to Figure 2)

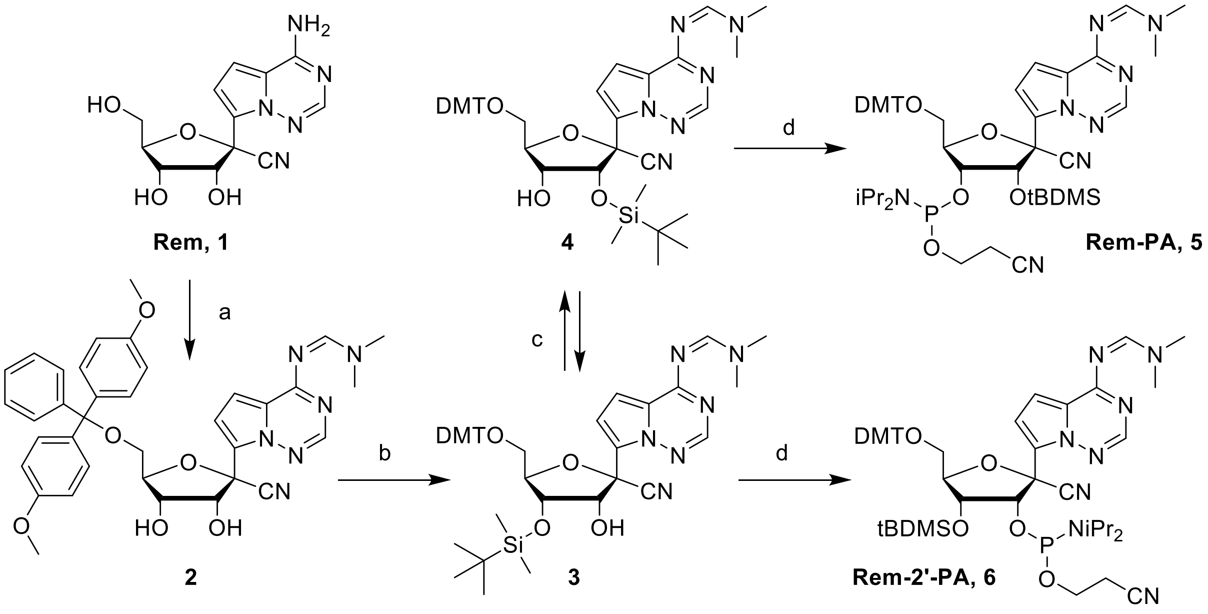

Supplementary Figure 4. Synthesis of Rem-PA. a) i) DMFDMA, pyridine, rt, 18 h, ii) DMT-Cl, pyridine, rt, 3.5 h, iii) MeOH, rt, 30 min, 93%; b) tBDMS-Cl, $AgNO_3$, pyridine, rt, 22 h, 72%; c) MeOH, Et_3N, rt, 30 min, 30% (**4**); d) CEPCl, $EtNMe_2$, DCM, rt, 5 h, 75% (**5**), 85% (**6**).

1'-Cyano-4-aza-7,9-dideazaadenosine (Rem, **1**) was reacted with dimethylformamide dimethylacetal (DMFDMA) in pyridine, resulting in protection of N^6 and temporary 2',3'-acetal formation. Installation of the 5'-*O*-4,4'-dimethoxytrityl group was followed by release of the 2',3'-acetal and isolation of compound **2**. Treatment with tBDMS-Cl in the presence of $AgNO_3$ produced predominantly the 3'-*O*-tBDMS-protected nucleoside **3**, which was equilibrated in MeOH/NEt_3 to allow isolation of the 2'-*O*-tBDMS-protected isomer **4**. Both compounds **3** and **4** were individually converted to the corresponding 2-cyanoethyl diisopropylphosphoramidites **5** and **6**, which were used in solid-phase synthesis of RNA oligonucleotides. Compound **5** was used for internal incorporation of RMP at positions –3 (R-3) and –4 (R-4), and compound **6** was used for synthesis of RNA containing Rem at the 3'-end (R-1).

Supplementary Table 2. Sequences and high-resolution ESI-MS data of synthetic RNA oligonucleotides.

Name	5'-sequence-3'	comment	nt	Mass calc.	Mass found
R-1	UGAGCCUACGCGR	Prepared with **6**	13	4241.574 Da	4241.570 Da
R-3	UGAGCCUACGCGRUG	Prepared with **5**	15	4812.680 Da	4812.702 Da
R-4	UGAGCCUACGCRGUG	Prepared with **5**	15	4812.680 Da	4812.707 Da
A-4	UGAGCCUACGCAGUG	Unmodified RNA	15	4788.680 Da	4788.702 Da

1'-Cyano-5'-*O*-(4,4'-Dimethoxytrityl)-N^6-dimethylformamidine-4-aza-7,9-dideazaadenosine (**2**)

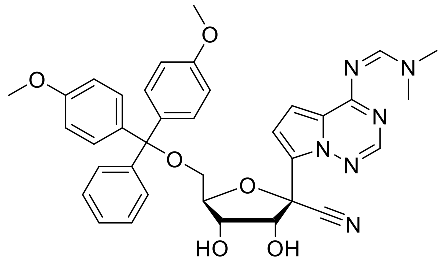

2
$C_{36}H_{36}N_6O_6$
648.72 g•mol^{-1}

A suspension of 1'-Cyano-4-aza-7,9-dideazaadenosine (**1**, 400 mg, 1.37 mmol, 1.00 eq.) in dry pyridine (4 mL) was treated with *N*,*N*-dimethylformamide dimethyl acetal (550 µL, 4.12 mmol, 3.00 eq.) and stirred at ambient temperature for 18 h. Volatiles were removed *in vacuo* and the residue was redissolved in dry pyridine (4 mL). 4,4'-Dimethoxytrityl chloride (560 mg, 1.65 mmol, 1.20 eq.) was added. The resulting solution was stirred at ambient temperature for 3.5 h. Methanol (16 mL) was added and stirring was continued for 30 min. Volatiles were removed under reduced pressure and the residue was purified by column chromatography (silica gel, CH_2Cl_2:MeOH 98:2 + 2% Et_3N) to yield the product **2** as a white foam (830 mg, 1.28 mmol, 93%).

TLC (silica gel, CH_2Cl_2:MeOH 98:2 + 2% Et_3N): R_f = 0.40.

^{1}H NMR (400 MHz, $CDCl_3$): δ (ppm) = 8.89 (br, 1H, N6-CH), 8.09 (s, 1H, C2-H), 7.27 – 7.23 (m, 2H, trityl-H), 7.21 – 7.13 (m, 7H, trityl-H), 7.04 (d, *J* = 4.6 Hz, 1H, C7-H), 7.00 (d, *J* = 4.6 Hz, 1H, C8-H), 6.77 – 6.70 (m, 4H, trityl-H), 4.78 (d, *J* = 5.3 Hz, 1H, C2'-H), 4.60 (td, *J* = 3.1, 1.7 Hz, 1H, C4'-H), 4.35 (dd, *J* = 5.3, 1.7 Hz, 1H, C3'-H), 3.77 (s, 3H, trityl-OCH_3), 3.75 (s, 3H, trityl-OCH_3), 3.50 (dd, *J* = 10.6, 3.1 Hz, 1H, C5'-H^a), 3.27 (d, J = 0.7 Hz, 3H, NCH_3^a), 3.25 (d, J = 0.5 Hz, 3H, NCH_3^b), 3.14 (dd, J = 10.6, 3.1 Hz, 1H, C5'-H^b).

^{13}C{^{1}H} NMR (100 MHz, $CDCl_3$): δ (ppm) = 161.07 (1C, C6), 158.56 (2C, trityl-C), 158.08 (1C, N6-CH), 147.91 (1C, C2), 144.57 (1C, trityl-C), 135.87 (1C, trityl-C), 135.51 (1C, trityl-C), 130.13 (2C, trityl-C), 130.09 (2C, trityl-C), 128.14 (2C, trityl-C), 127.91 (2C, trityl-C), 126.90 (1C, trityl-C), 125.69 (1C, C9), 122.53 (1C, C5), 116.78 (1C, CN), 113.23 (2C, trityl-C), 113.20 (2C, trityl-C), 111.46 (1C, C7), 103.87 (1C, C8), 87.20 (1C, C4'), 86.43 (1C, trityl-C), 79.24 (1C, C1'), 77.11 (1C, C2'), 73.49 (1C, C3'), 63.44 (1C, C5'), 55.33 (2C, trityl-OCH_3), 55.31 (2C, trityl-OCH_3), 41.76 (NCH_3^a), 35.54 (NCH_3^b).

HR-ESI-MS: *m*/*z* calc. ($C_{36}H_{37}N_6O_6$ $[M+H]^+$): 649.27691, found: 649.27836.

1'-Cyano-5'-*O*-(4,4'-Dimethoxytrityl)-*N*[6]-dimethylformamidine-3'-*O*-(*tert*-butyldimethylsilyl)-4-aza-7,9-dideazaadenosine (**3**)

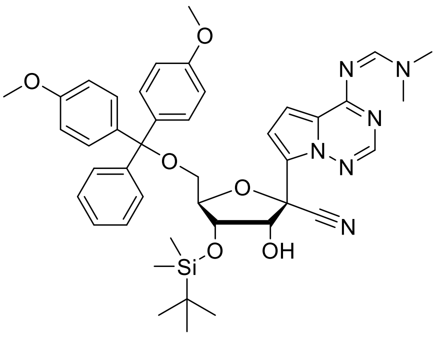

3
$C_{42}H_{50}N_6O_6Si$
762.98 g•mol^{-1}

A solution of **2** (200 mg, 308 µmol, 1.00 eq.) in dry pyridine (2 mL) was treated with silver nitrate (209 mg, 1.23 mmol, 4.00 eq.) and stirred in the dark at ambient temperature for 30 min. *tert*-Butyldimethylsilyl chloride (55.8 mg, 370 µmol, 1.20 eq.) was added and stirring was continued in the dark for 22 h. Volatiles were removed under reduced pressure. The residue was taken up in ethyl acetate and insoluble residues were removed by filtration through a pad of Celite. The filtrate was evaporated to dryness and the residue was purified by column chromatography (silica gel, *n*-hexane:EtOAc 1:1 + 1% Et_3N to 0:1 + 1% Et_3N) to yield the product **3** as a white foam (169 mg, 221 µmol, 72%).

TLC (silica gel, *n*-hexane:EtOAc 1:3): R_f = 0.31.

^{1}H NMR (400 MHz, $CDCl_3$): δ (ppm) = 8.83 (br, 1H, N6-CH), 8.04 (s, 1H, C2-H), 7.45 – 7.13 (m, 9H, trityl-H), 7.05 (d, *J* = 4.6 Hz, 1H, C7-H), 6.93 (d, *J* = 4.6 Hz, 1H, C8-H), 6.79 – 6.72 (m, 4H, trityl-H), 5.08 (dd, *J* = 9.1, 5.5 Hz, 1H, C2'-H), 4.39 (m, 1H, C4'-H), 4.34 (dd, *J* = 5.5, 2.3 Hz, 1H, C3'-H), 3.78 – 3.75 (m, 7H, (trityl-$OCH_3)_2$, C2'-OH), 3.50 (dd, *J* = 10.6, 4.2 Hz, 1H, C5'-H^a), 3.25 (d, *J* = 0.6 Hz, 3H, $NCH_3{}^a$), 3.22 (d, *J* = 0.5 Hz, 3H, $NCH_3{}^b$), 3.16 (dd, *J* = 10.6, 3.4 Hz, 1H, C5'-H^b), 0.93 (s, 9H, Si-$C(CH_3)_3$), 0.09 (s, 3H, Si-CH_3), 0.00 (s, 3H, Si-CH_3).

^{13}C{^{1}H} NMR (100 MHz, $CDCl_3$): δ (ppm) = 160.73 (1C, C6), 158.46 (2C, trityl-C), 157.55 (1C, N6-CH), 147.38 (1C, C2), 144.51 (1C, trityl-C), 135.84 (1C, trityl-C), 135.59 (1C, trityl-C), 130.02 (4C, trityl-C), 128.15 (2C, trityl-C), 127.79 (2C, trityl-C), 126.80 (1C, trityl-C), 123.60 (1C, C7), 123.45 (1C, C5), 116.53 (1C, CN), 113.08 (4C, trityl-C), 112.69 (1C, C9), 102.52 (1C, C8), 86.38 (1C, trityl-C), 86.21 (1C, C4'), 78.73 (1C, C1'), 74.69 (1C, C2'), 72.77 (1C, C3'), 62.53 (1C, C5'), 55.20 (2C, trityl-OCH_3), 41.46 (1C, $NCH_3{}^a$), 35.27 (1C, NCH^b), 25.61 (3C, Si-$C(\underline{C}H_3)_3$), 17.99 (1C, Si-$\underline{C}(CH_3)_3$), -4.63 (1C, $SiCH_3$), -4.99 (1C, Si-CH_3).

HR-ESI-MS: *m*/*z* calc. ($C_{42}H_{51}N_6O_6Si$ $[M+H]^+$): 763.36339, found: 763.36464.

1'-Cyano-5'-*O*-(4,4'-Dimethoxytrityl)-*N*[6]-dimethylformamidine-2'-*O*-(*tert*-butyldimethylsilyl)-4-aza-7,9-dideazaadenosine (**4**)

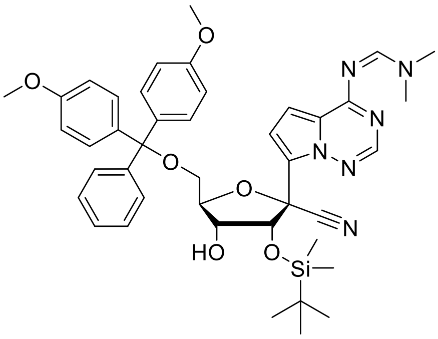

4

$C_{42}H_{50}N_6O_6Si$

762.98 g•mol^{-1}

Compound **3** (100 mg, 131 µmol, 1.00 eq.) was dissolved in a mixture of methanol (99 mL) and triethylamine (1 mL). After stirring at ambient temperature for 30 min the ratio of isomers **3** and **4** was ca. 1:1 (by TLC). After removal of the solvent under reduced pressure and purification of the residue by column chromatography (*n*-hexane:EtOAc 1:3) 30.0 mg (39.3 µmol, 30%) of pure product **4** were obtained as a white foam. The remaining mixture of isomers was isolated and again treated with trimethylamine in methanol.

TLC (silica gel, *n*-hexane:EtOAc 1:3): R_f = 0.41.

^{1}H NMR (400 MHz, $CDCl_3$): δ (ppm) = 8.85 – 8.80 (m, 1H, N6-CH), 7.79 (s, 1H, C2-H), 7.51 – 7.43 (m, 2H, trityl-H), 7.39 – 7.32 (m, 4H, trityl-H), 7.28 – 7.15 (m, 3H, trityl-H), 7.11 (d, *J* = 4.6 Hz, 1H, C7-H), 6.91 (d, *J* = 4.6 Hz, 1H, C8-H), 6.82 – 6.73 (m, 4H, trityl-H), 5.50 (d, *J* = 5.7 Hz, 1H, C2'-H), 4.45 (m, 1H, C4'-H), 4.34 (m, 1H, C3'-H), 3.77 (2s, 6H, trityl-OCH_3), 3.50 (dd, *J* = 10.4, 4.0 Hz, 1H, C5'-H[a]), 3.34 (dd, *J* = 10.4, 4.4 Hz, 1H, C5'-H[b]), 3.26 (d, *J* = 0.7 Hz, 3H, NCH_3), 3.22 (d, *J* = 0.4 Hz, 3H, NCH_3), 2.77 (d, *J* = 4.3 Hz, 1H, C3'-OH), 0.87 (s, 9H, Si-$C(CH_3)_3$), -0.10 (s, 3H, Si-CH_3), -0.15 (s, 3H, Si-CH_3).

^{13}C{^{1}H} NMR (100 MHz, $CDCl_3$): δ (ppm) = 160.83 (1C, C6), 158.55 (1C, trityl-C), 158.53 (1C, trityl-C), 157.71 (1C, N6-CH), 147.20 (1C, C2), 145.03 (1C, trityl-C), 136.22 (1C, trityl-C), 136.11 (1C, trityl-C), 130.28 (4C, trityl-C), 128.41 (2C, trityl-C), 127.87 (2C, trityl-C), 126.85 (1C, trityl-C), 123.86 (1C, C5), 122.29 (1C, C7), 117.10 (1C, CN), 114.81 (1C, C9), 113.18 (4C, trityl-C), 102.52 (1C, C8), 86.25 (1C, trityl-C), 85.43 (1C, C4'), 80.06 (1C, C1'), 73.61 (1C, C2'), 71.68 (1C, C3'), 63.02 (1C, C5'), 55.34 (2C, trityl-OCH_3), 41.62 (1C, NCH_3), 35.42 (1C, NCH_3), 25.75 (3C, Si-$C(\underline{C}H_3)_3$), 18.11 (1C, Si-$\underline{C}(CH_3)_3$), -4.91 (1C, Si-CH_3), -5.12 (1C, Si-CH_3).

HR-ESI-MS: *m*/*z* calc. ($C_{42}H_{51}N_6O_6Si$ $[M+H]^+$): 763.36339, found: 763.36413.

1'-Cyano-5'-*O*-(4,4'-Dimethoxytrityl)-N^6-dimethylformamidine-2'-*O*-(*tert*-butyldimethylsilyl)-4-aza-7,9-dideazaadenosine 3'-β-cyanoethyl diisopropyl phosphoramidite (**5**)

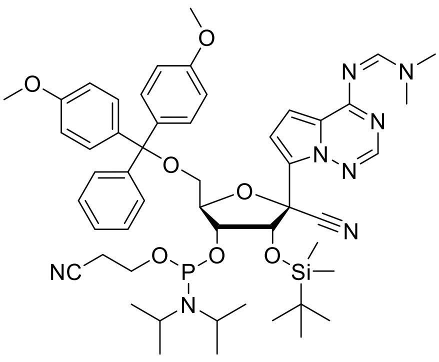

5

$C_{51}H_{67}N_8O_7PSi$

963.20 g•mol^{-1}

A solution of **4** (110.0 mg, 144 µmol, 1.00 eq.) in dry dichloromethane (1.1 mL) was treated with *N*,*N*-dimethylethylamine (156 µL, 1.44 mmol, 10.0 eq.) and 2-cyanoethyl *N*,*N*-diisopropyl-chlorophosphoramidite (40.9 mg, 173 µmol, 1.20 eq.) and stirred at ambient temperature for 5 h. Volatiles were removed under reduced pressure and the residue was purified by column chromatography (silica gel, *n*-hexane:EtOAc 1:3) to yield the desired product as a white foam (104 mg, 108 µmol, 75%, isomer ratio at phosphorus 10:8).

TLC (silica gel, *n*-hexane:EtOAc 1:3): R_f = 0.45.

^{1}H NMR (400 MHz, $CDCl_3$): δ (ppm) = 8.82, 8.81 (2 s, N6-CH), 7.68 (s, C2-H), 7.58 (s, C2-H), 7.54 – 7.45 (m, trityl-H), 7.43 – 7.33 (m, trityl-H), 7.27 – 7.18 (m, trityl-H), 7.16, 7.15 (2 d, *J* = 4.6 Hz, C7-H), 6.92, 6.91 (2 d, *J* = 4.6 Hz, C8-H), 6.83 – 6.72 (m, trityl-H), 5.63, 5.61 (2 d, *J* = 5.3 Hz, C2'-H), 4.62 (app q, *J* = 3.3 Hz, C4'-H), 4.58 – 4.51 (m, C4'-H), 4.45 – 4.34 (m, C3'-H), 4.20 – 4.09 (m, $POCH_2$), 3.98 – 3.88 (m, $POCH_2$), 3.78 – 3.75 (m, trityl-OCH_3), 3.66 – 3.52 (m, C5'-H, $N(C\underline{H}(CH_3)_2)_2$), 3.34 (dd, *J* = 10.3, 4.5 Hz, C5'-H), 3.28 – 3.23 (m, C5'-H, $N(CH_3)_2$), 3.21 (d, *J* = 0.9 Hz, $N(CH_3)_2$), 2.73 – 2.54 (m, CH_2CN), 2.24 – 2.18 (m, CH_2CN), 1.18 (d, *J* = 6.8 Hz, $N(CH(C\underline{H}_3)_2)_2$), 1.15 (d, *J* = 6.8 Hz, $N(CH(C\underline{H}_3)_2)_2$), 1.00 (d, *J* = 6.8 Hz, $N(CH(C\underline{H}_3)_2)_2$), 0.80 (s, 4H), 0.77 (s, $SiCH_3$), -0.09 (s, $SiCH_3$), -0.18 (s, $SiCH_3$), -0.33 (s, $SiCH_3$), -0.40 (s, $SiCH_3$).

^{13}C{^{1}H} NMR (100 MHz, $CDCl_3$): δ (ppm) = 160.79 (C6), 158.55 (trityl-C), 157.67 (N6-CH), 147.07 (C2), 146.98 (C2), 145.05 (trityl-C), 144.87 (trityl-C), 136.29 (trityl-C), 136.17 (trityl-C), 136.09 (trityl-C), 136.02 (trityl-C), 130.41 (trityl-C), 130.36 (trityl-C), 128.57 (trityl-C), 128.52 (trityl-C), 127.85 (trityl-C), 126.88 (trityl-C), 123.98 (C5), 123.81 (C5), 122.73 (C9), 122.22 (C9), 118.34 ($CH_2\underline{C}N$), 117.95 (C1'-$\underline{C}$N), 117.53 ($CH_2\underline{C}N$), 117.32 (C1'-$\underline{C}$N), 116.00 (C7), 115.46 (C7), 113.15 (trityl-C), 113.13 (trityl-C), 102.51 (C8), 86.43 (trityl-C), 86.24 (trityl-C), 85.79 (C4'), 79.90 (C1'), 79.69 (C1'), 73.45 (C2'), 72.46 (C3'), 72.35 (C3'), 63.32 (C5'), 63.04 (C5'), 59.26 ($POCH_2$), 59.14 ($POCH_2$), 57.78 ($POCH_2$), 57.59 ($POCH_2$), 55.35 (trityl-OCH_3), 43.55 ($N\underline{C}H(CH_3)_2$), 43.43 ($N\underline{C}H(CH_3)_2$), 43.04 ($N\underline{C}H(CH_3)_2$), 42.91 ($N\underline{C}H(CH_3)_2$), 41.59 (NCH_3), 35.41 (NCH_3), 25.87 ($SiCH(\underline{C}H_3)_3$) 25.85 ($SiCH(\underline{C}H_3)_3$), 25.81 ($SiCH(\underline{C}H_3)_3$), 24.93 ($NCH(\underline{C}H_3)_2$), 24.85 ($NCH(\underline{C}H_3)_2$), 24.75 ($NCH(\underline{C}H_3)_2$), 24.69 ($NCH(\underline{C}H_3)_2$), 24.63 ($NCH(\underline{C}H_3)_2$), 20.88 ($\underline{C}H_2$-CN), 20.83 ($\underline{C}H_2$-CN), 18.11 ($Si\underline{C}H(CH_3)_3$), 18.04 ($Si\underline{C}H(CH_3)_3$), -4.64 ($SiCH_3$), -4.68 ($SiCH_3$), -5.37 ($SiCH_3$), -5.45 ($SiCH_3$).

^{31}P{^{1}H} NMR (162 MHz, $CDCl_3$): δ (ppm) = 150.48, 147.94.

HR-ESI-MS: *m/z* calc. ($C_{51}H_{68}N_8O_7PSi$ $[M+H]^+$): 963.47124, found: 963.46940.

1'-Cyano-5'-*O*-(4,4'-Dimethoxytrityl)-*N*^{6}-dimethylformamidine-3'-*O*-(*tert*-butyldimethylsilyl)-4-aza-7,9-dideazaadenosine 2'-β-cyanoethyl diisopropyl phosphoramidite (**6**)

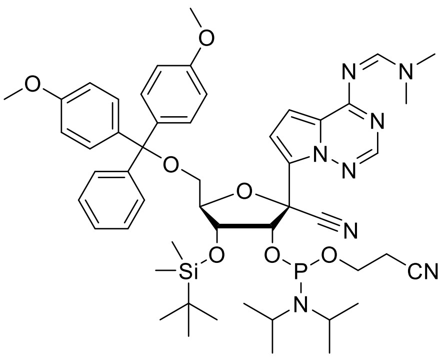

6
$C_{51}H_{67}N_8O_7PSi$
963.20 g•mol^{-1}

A solution of **3** (50.0 mg, 65.5 µmol, 1.00 eq.) in dry dichloromethane (0.5 mL) was treated with *N*,*N*-dimethylethylamine (71.0 µL, 655 µmol, 10.0 eq.) and 2-cyanoethyl *N*,*N*-diisopropyl-chlorophosphoramidite (18.6 mg, 78.6 µmol, 1.20 eq.) and stirred at ambient temperature for 5 h. Volatiles were removed under reduced pressure and the residue was purified by column chromatography (silica gel, *n*-hexane:EtOAc 1:2) to yield the product as a white foam (54.0 mg, 56.1 µmol, 85%, isomer ratio at phosphorus 10:3).

TLC (silica gel, *n*-hexane:EtOAc 1:2): R_f = 0.20.

^{1}H NMR (400 MHz, $CDCl_3$): δ (ppm) = 8.82 (s, N6-CH), 8.79 (t, *J* = 0.7 Hz, N6-CH), 7.92 (s, C2-H), 7.72 (s, C2-H), 7.46 – 7.16 (m, C7-H, trityl-H), 7.13 (dd, *J* = 4.6, 0.8 Hz, C7-H), 6.91 (d, *J* = 4.6 Hz, C8-H), 6.86 (d, *J* = 4.6 Hz, C8-H), 6.82 – 6.74 (m, trityl-H), 5.37 (dd, *J* = 12.6, 4.7 Hz, 1H, C2'-H), 5.28 (dd, *J* = 10.8, 4.1 Hz, 1H, C2'-H), 4.48 – 4.37 (m, 3'-H, 4'-H), 3.90 – 3.82 (m, $POCH_2$), 3.82 – 3.72 (m, (trityl-$OCH_3)_2$, $POCH_2$), 3.69 – 3.47 (m, $POCH_2$, N(C<u>H</u>$(CH_3)_2)_2$, C5'-H), 3.30 (dd, *J* = 10.2, 4.1 Hz, C5'-H), 3.26 – 3.19 (m, $N(CH_3)_2$, C5'-H), 2.78 – 2.31 (m, CH_2CN), 1.11 (d, *J* = 6.8 Hz, N(CH(C<u>H</u>$_3)_2)_2$), 1.05 (d, *J* = 6.7 Hz, N(CH(C<u>H</u>$_3)_2)_2$), 1.02 – 0.94 (m, N(CH(C<u>H</u>$_3)_2)_2$, Si-C$(CH_3)_3$), 0.91 (s, Si-C$(CH_3)_3$), 0.14 (s, Si-CH_3), 0.12 (s, Si-CH_3), 0.09 (s, Si-CH_3), 0.00 (s, Si-CH_3).

^{13}C{^{1}H} NMR (100 MHz, $CDCl_3$): δ (ppm) = 160.78 (C6), 158.58 (trityl-C), 158.56 (trityl-C), 157.63 (N6-CH), 147.11 (C2), 144.82 (trityl-C), 136.07 (trityl-C), 135.92 (trityl-C), 130.28 (trityl-C), 130.24 (trityl-C), 130.16 (trityl-C), 128.46 (trityl-C), 128.40 (trityl-C), 127.93 (trityl-C), 127.89 (trityl-C), 126.92 (trityl-C), 123.65 (C5), 123.42 (C9), 117.75 (CH_2<u>C</u>N), 117.07 (C1'-<u>C</u>N), 114.03 (C7), 113.99 (C7), 113.21 (trityl-C), 113.19 (trityl-C), 102.44 (C8), 86.82 (trityl-C), 86.60 (trityl-C), 86.45 (C4'), 86.33 (C4'), 77.87 (C1'), 77.82 (C1'), 75.18 (C2'), 75.04 (C2'), 72.86 (C3'), 63.23 (C5'), 58.64 ($POCH_2$), 58.47 ($POCH_2$), 55.37 (trityl-OCH_3), 43.41 (N(<u>C</u>H$(CH_3)_2)_2$), 43.29 (N(<u>C</u>H$(CH_3)_2)_2$), 41.59 (NCH_3), 35.39 (NCH_3), 25.85 (Si-C(<u>C</u>H$_3)_3$), 24.83 (N(CH(<u>C</u>H$_3)_2)_2$), 24.76 (N(CH(<u>C</u>H$_3)_2)_2$), 24.69 (N(CH(<u>C</u>H$_3)_2)_2$), 24.61 (N(CH(<u>C</u>H$_3)_2)_2$), 24.39 (N(CH(<u>C</u>H$_3)_2)_2$), 24.32 (N(CH(<u>C</u>H$_3)_2)_2$), 20.46 (<u>C</u>H$_2$CN), 20.40 (<u>C</u>H$_2$CN), 18.11 (Si-<u>C</u>$(CH_3)_3$), -4.33 ($SiCH_3$), -4.36 ($SiCH_3$), -4.40 ($SiCH_3$), -4.42 ($SiCH_3$).

^{31}P{^{1}H} NMR (162 MHz, $CDCl_3$): δ (ppm) = 149.92, 149.25.

HR-ESI-MS: *m*/*z* calc. ($C_{51}H_{68}N_8O_7PSi$ $[M+H]^+$): 963.47124, found: 963.47136.

NMR Spectra

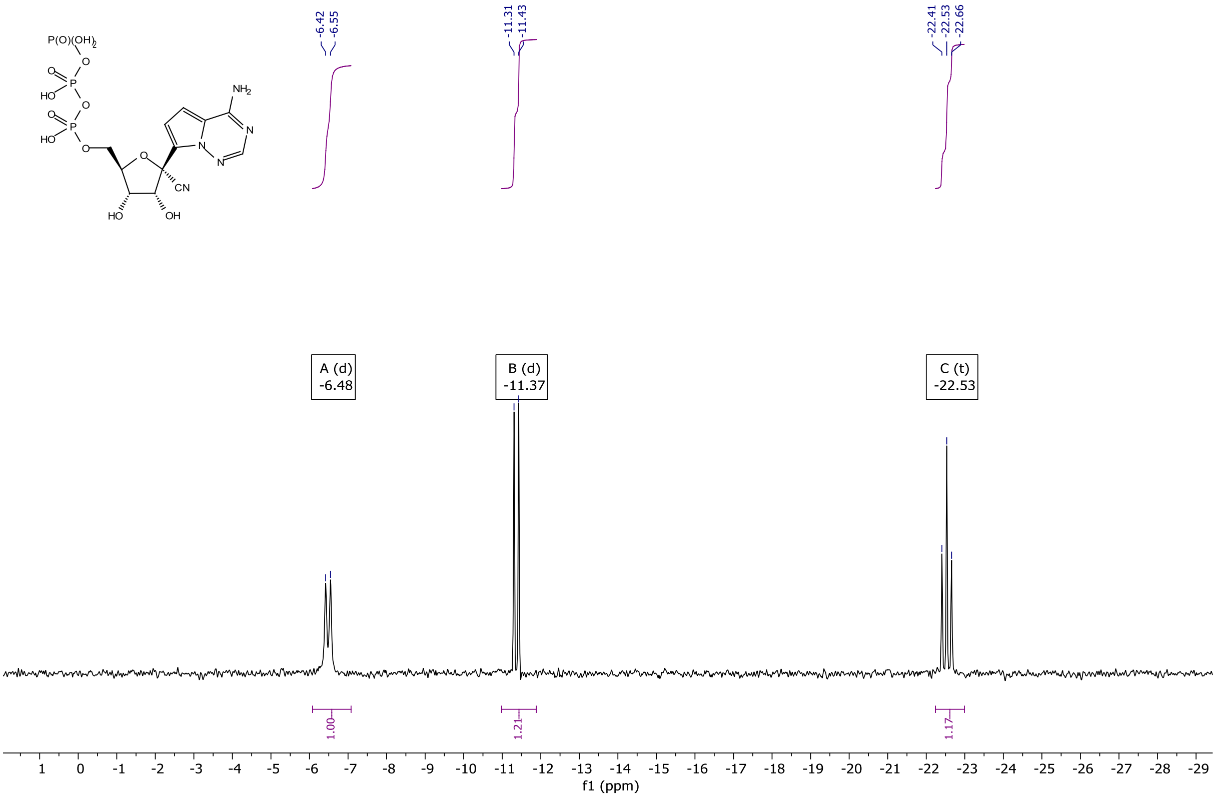

^{31}P NMR (162 MHz, D_2O) of **RTP***5$(C_2H_5)_3N$

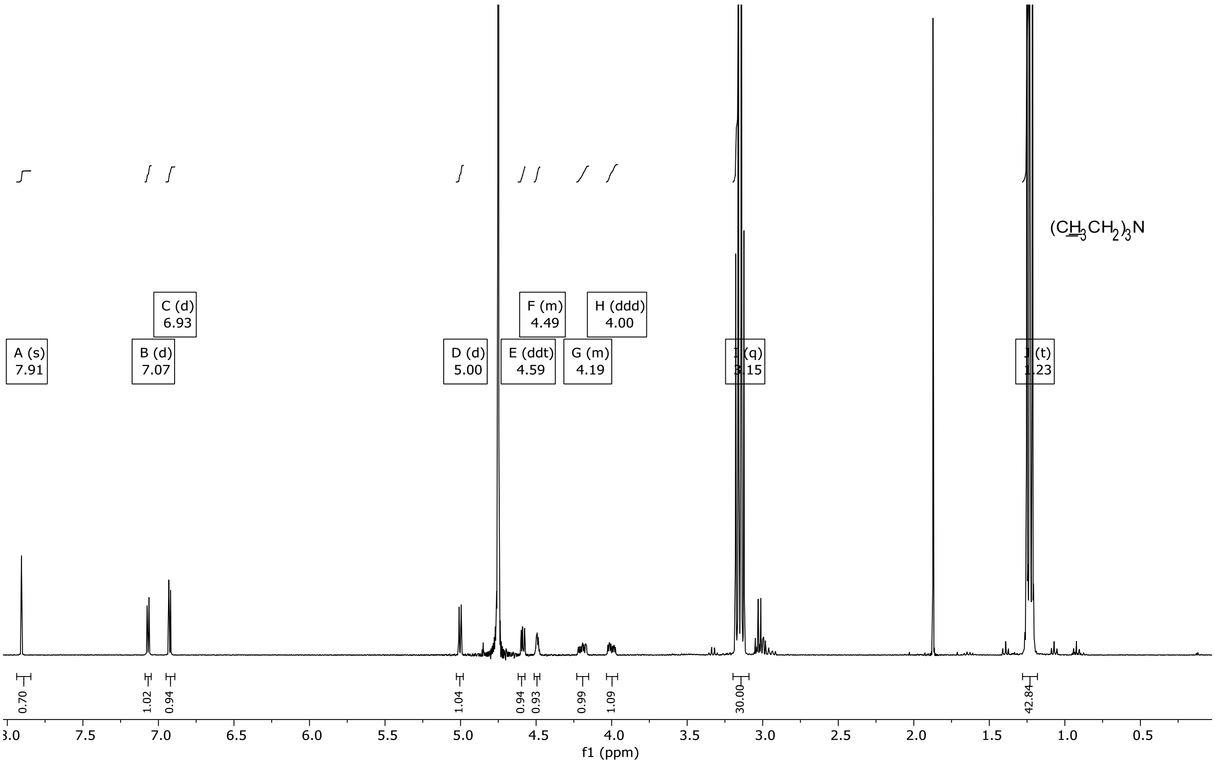

^{1}H NMR (400 MHz, D_2O) of **RTP*** 5$(C_2H_5)_3N$

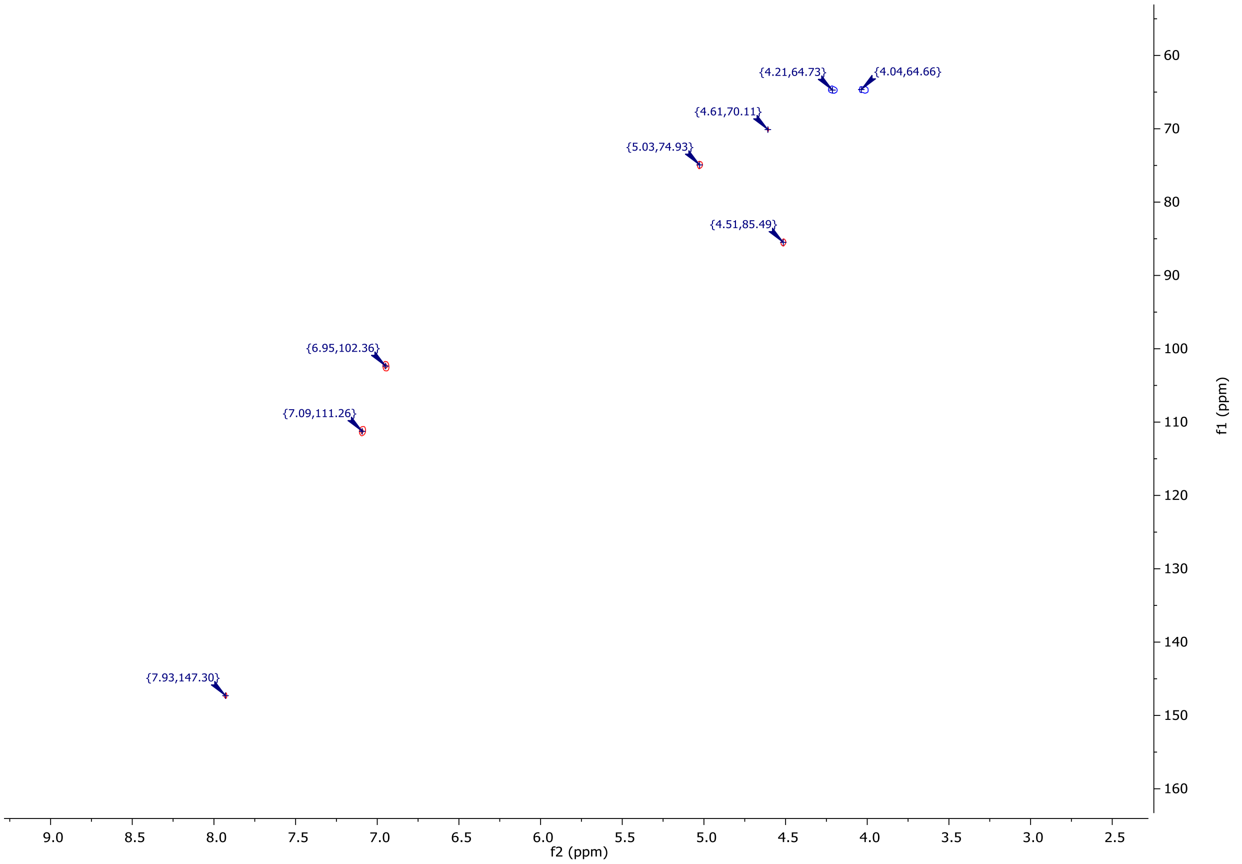

^{1}H /^{13}C-HSQC NMR (400 MHz, D_2O) of **RTP***5$(C_2H_5)_3N$

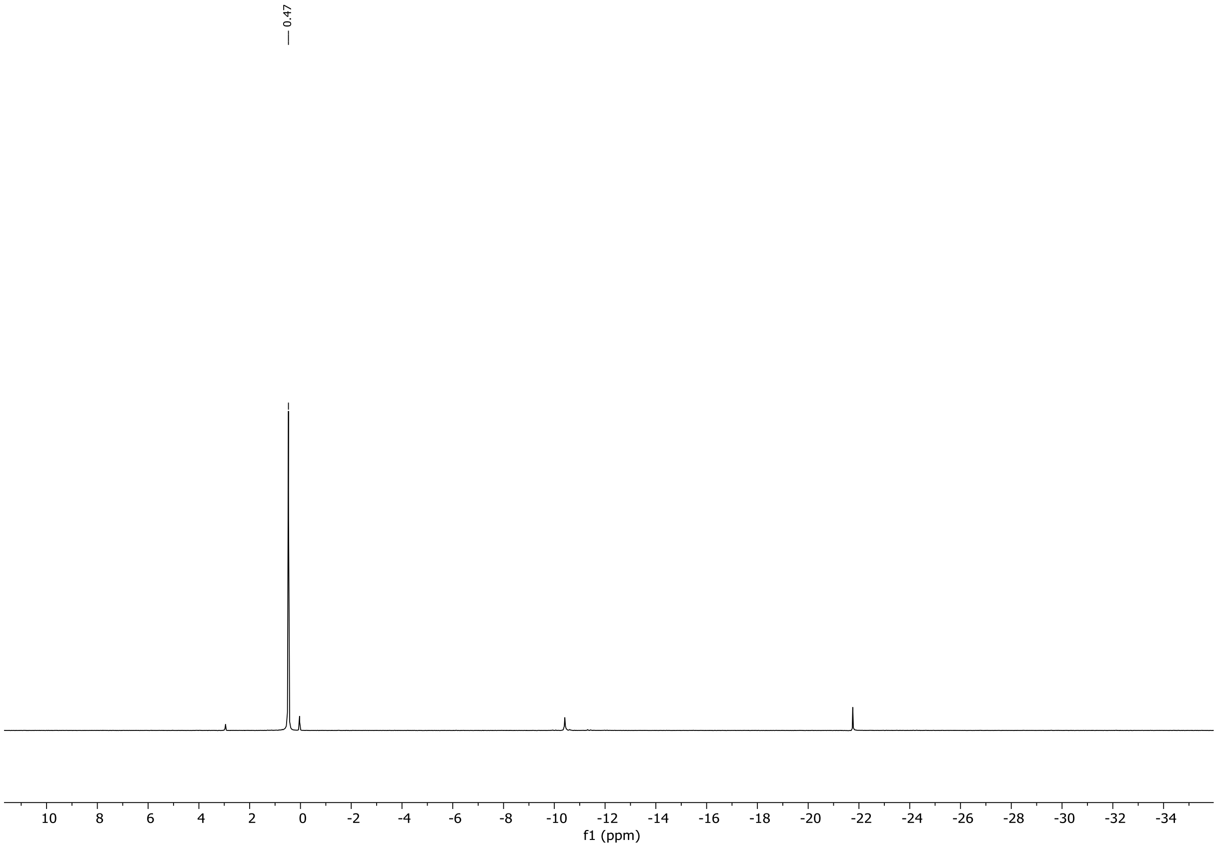

^{31}P NMR (162 MHz, D_2O) of **RMP**.

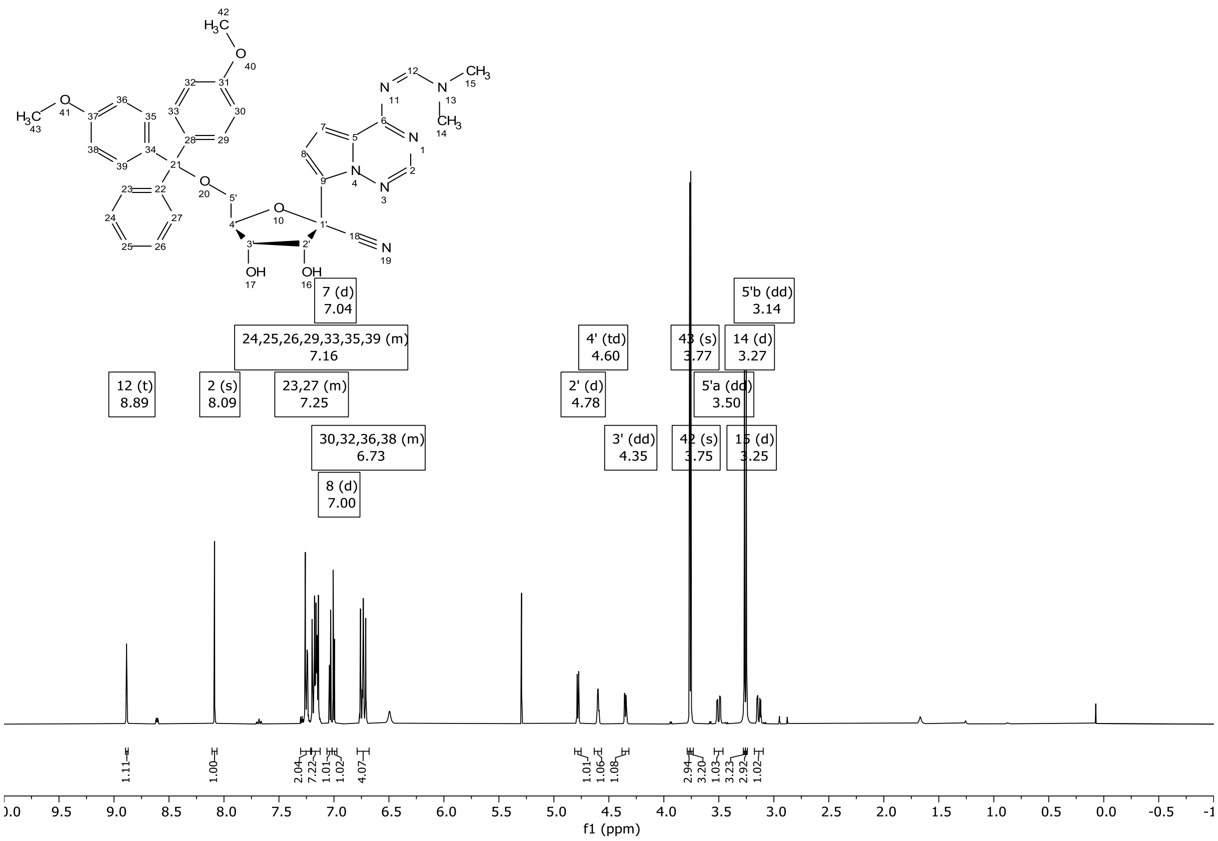

[1] H NMR (400 MHz, $CDCl_3$) of compound **2**.

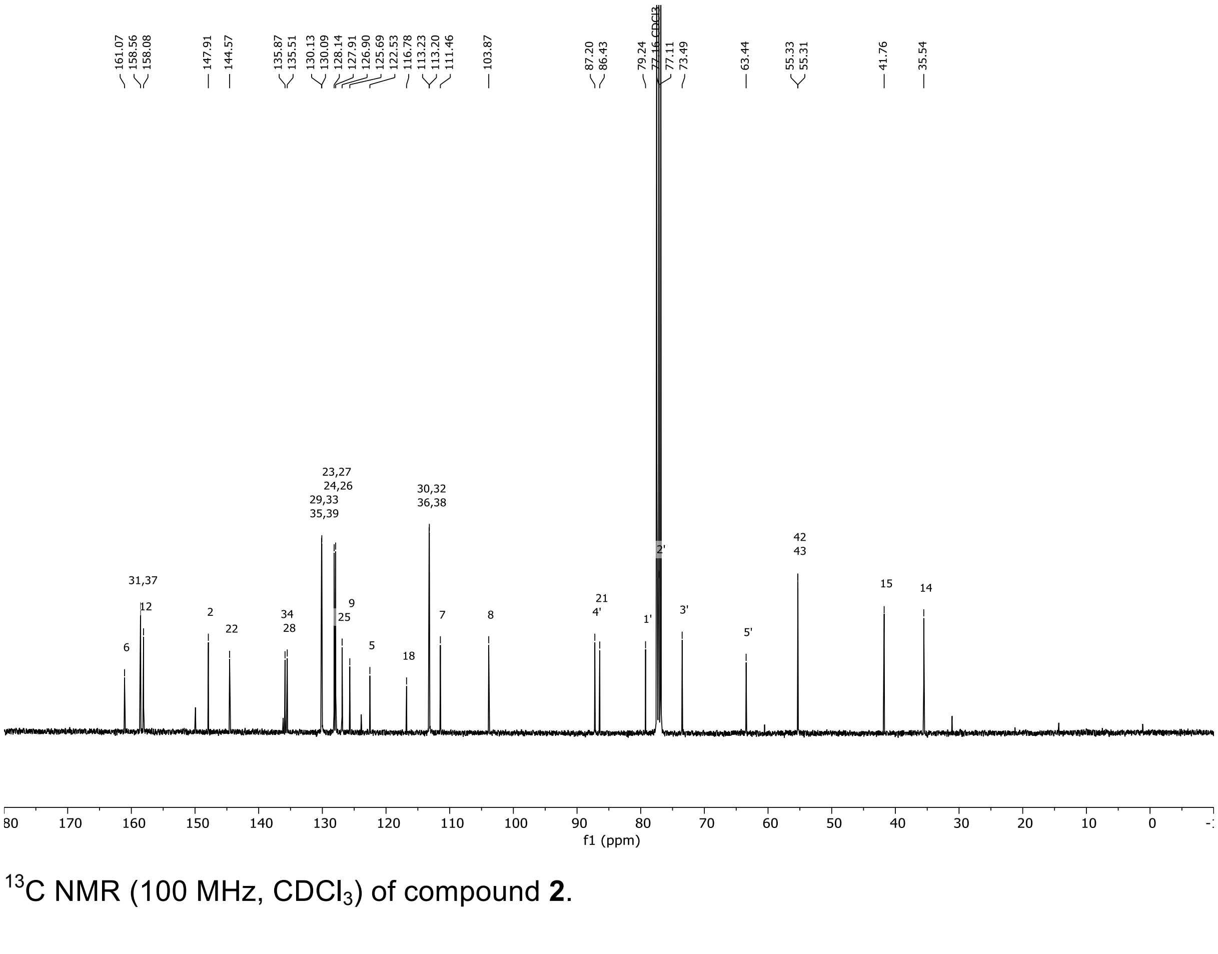

[13] C NMR (100 MHz, $CDCl_3$) of compound **2**.

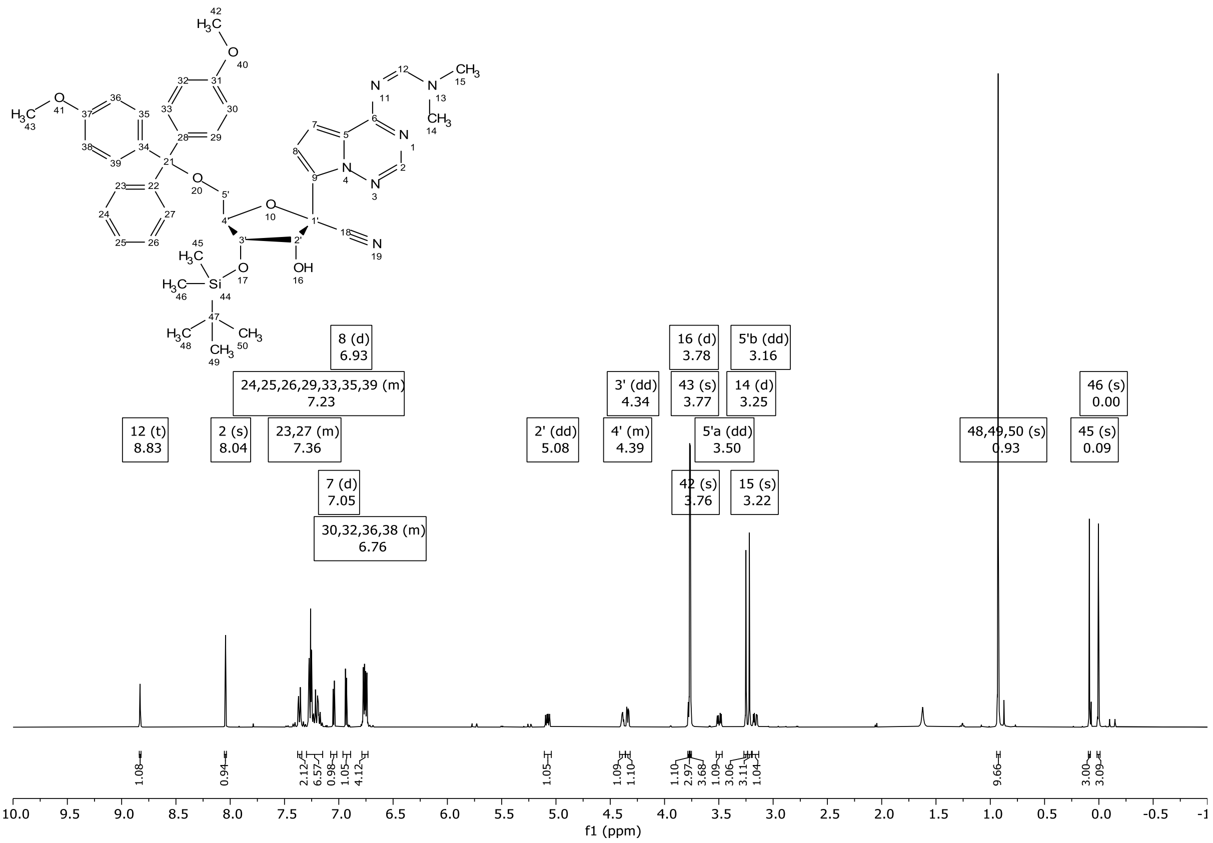

^1H NMR (400 MHz, $CDCl_3$) of compound **3**.

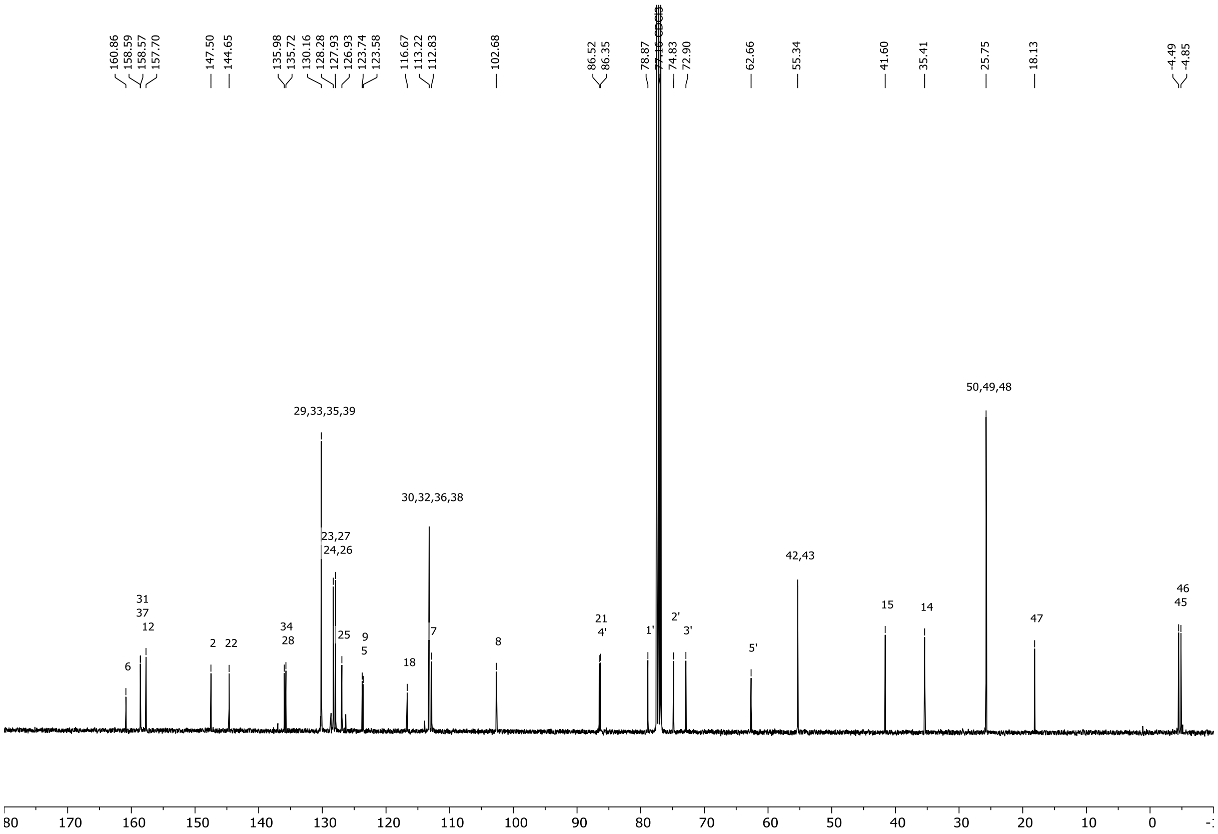

^{13}C NMR (100 MHz, $CDCl_3$) of compound **3**.

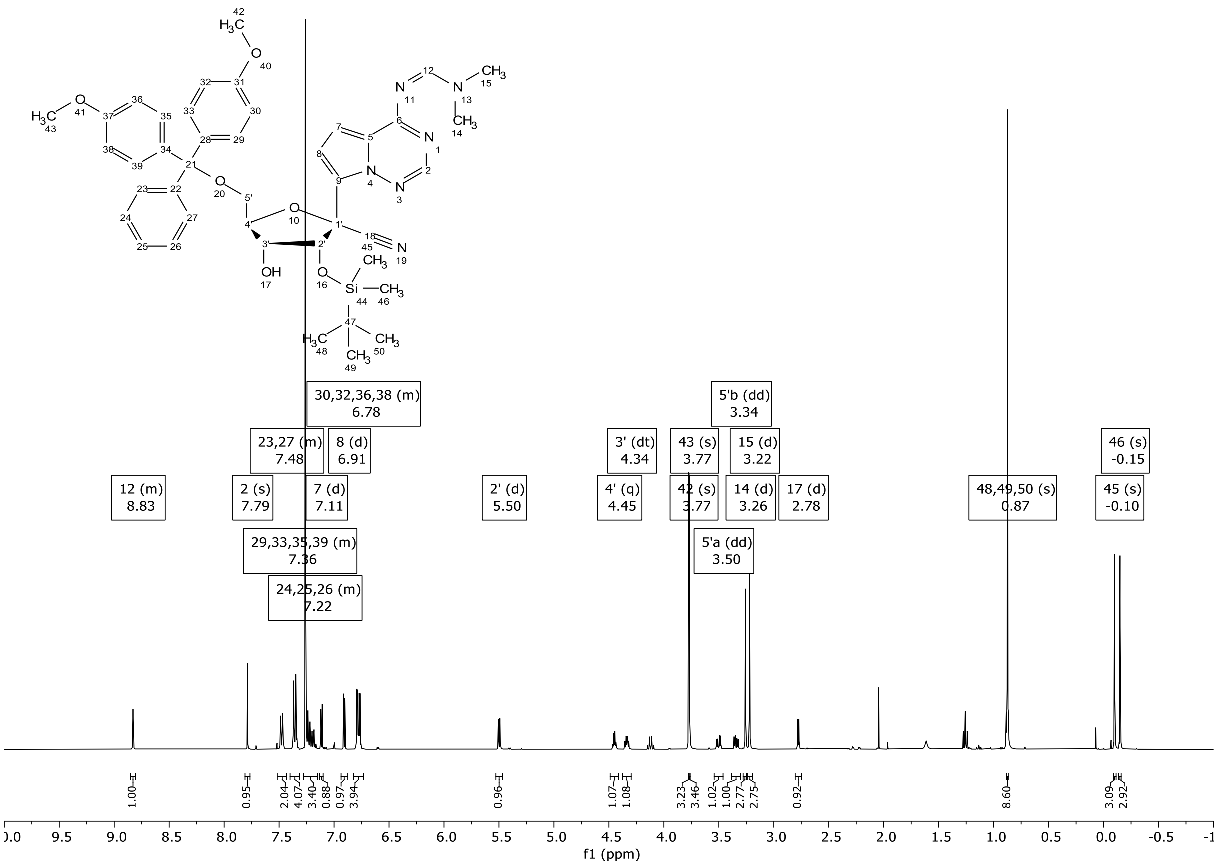

^{1}H NMR (400 MHz, $CDCl_3$) of compound **4**.

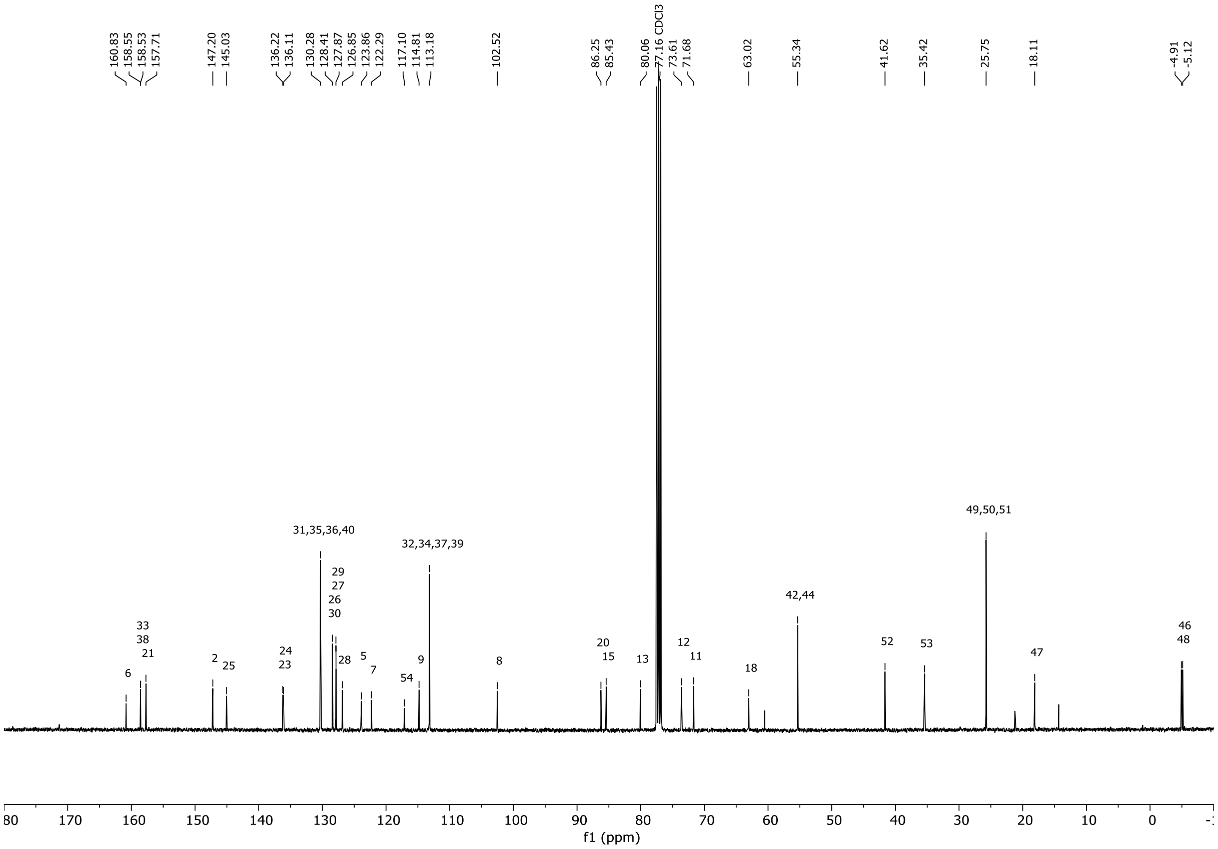

^{13}C NMR (100 MHz, $CDCl_3$)of compound **4**.

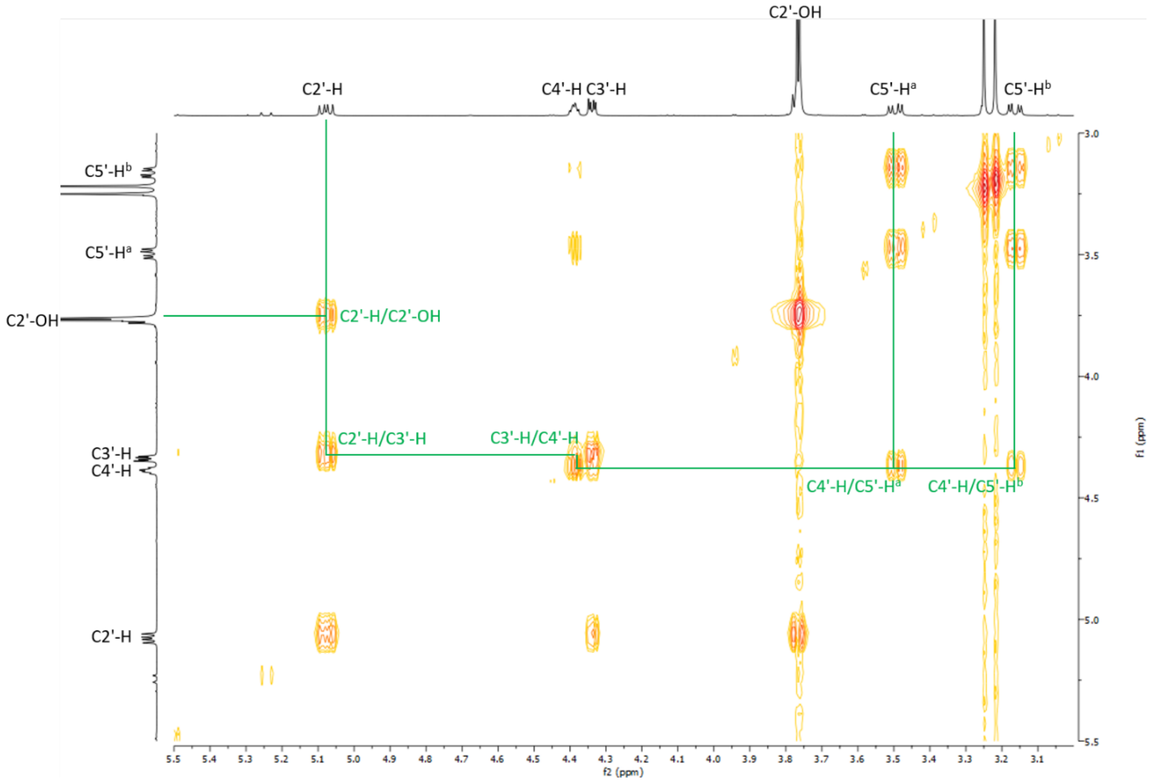

Excerpt of the ^{1}H/^{1}H-COSY NMR spectrum of compound **3**. The relevant cross-peaks displaying the connectivity of the ribose protons are highlighted. The presence of the C2'-H/C2'-OH cross-peak confirms that the TBDMS protecting group of **3** is attached to the C3'-OH.

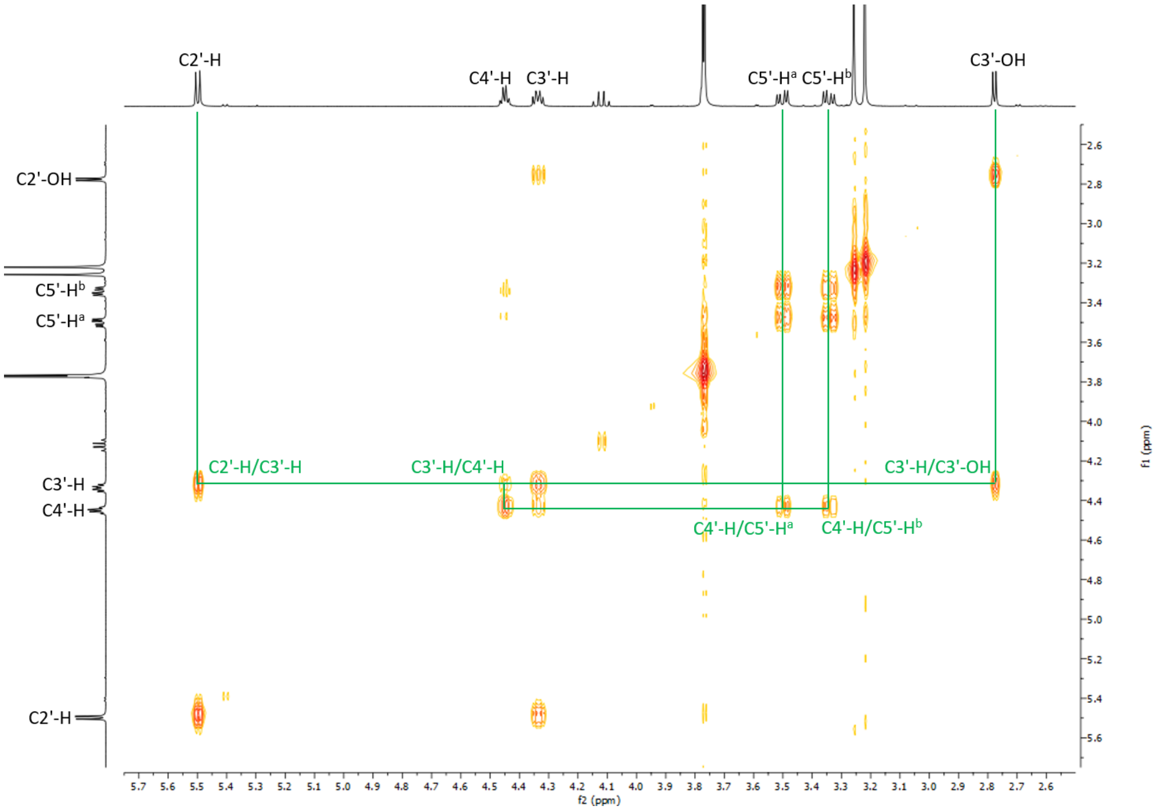

Excerpt of the ^{1}H/^{1}H-COSY NMR spectrum of compound **4**. The relevant cross-peaks displaying the connectivity of the ribose protons are highlighted. The presence of the C3'-H/C3'-OH cross-peak confirms that the TBDMS protecting group of **4** is attached to the C2'-OH.

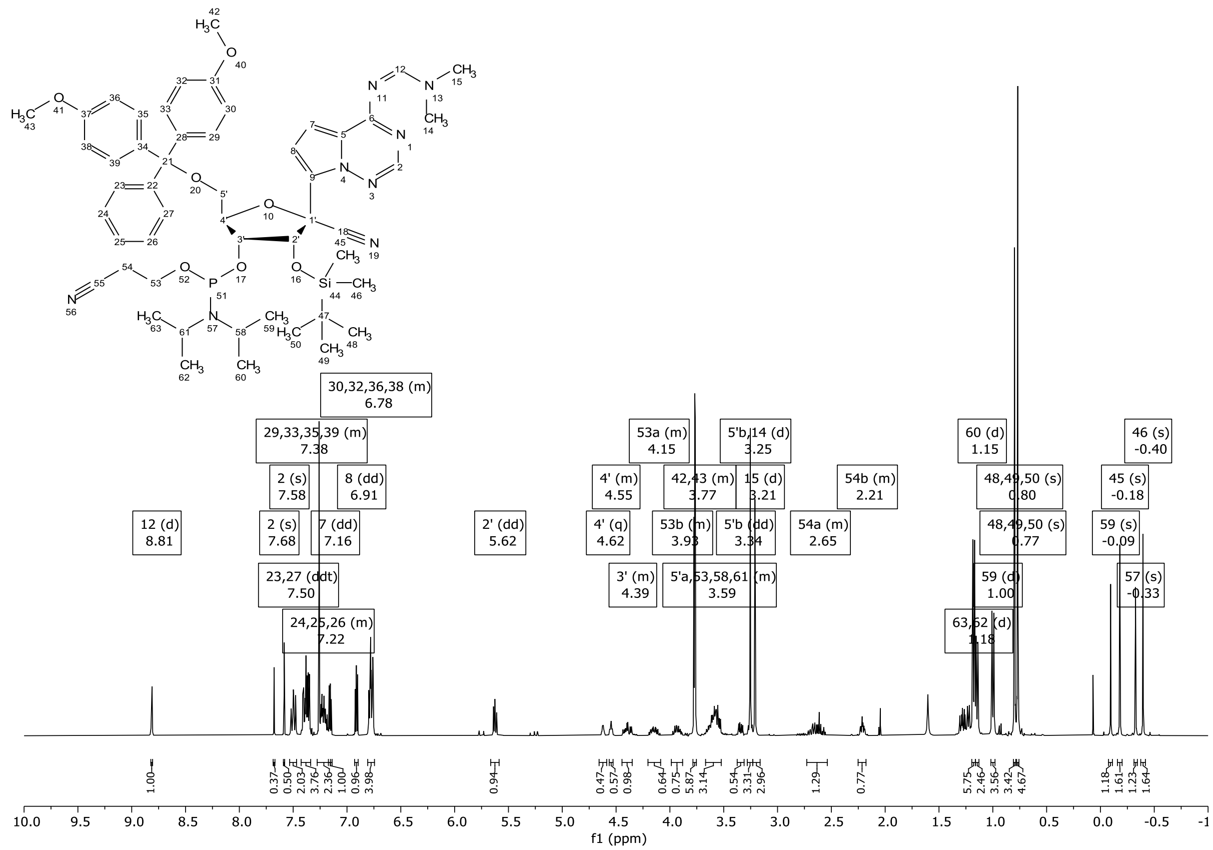

^{1}H NMR (400 MHz, $CDCl_3$) of compound **5**.

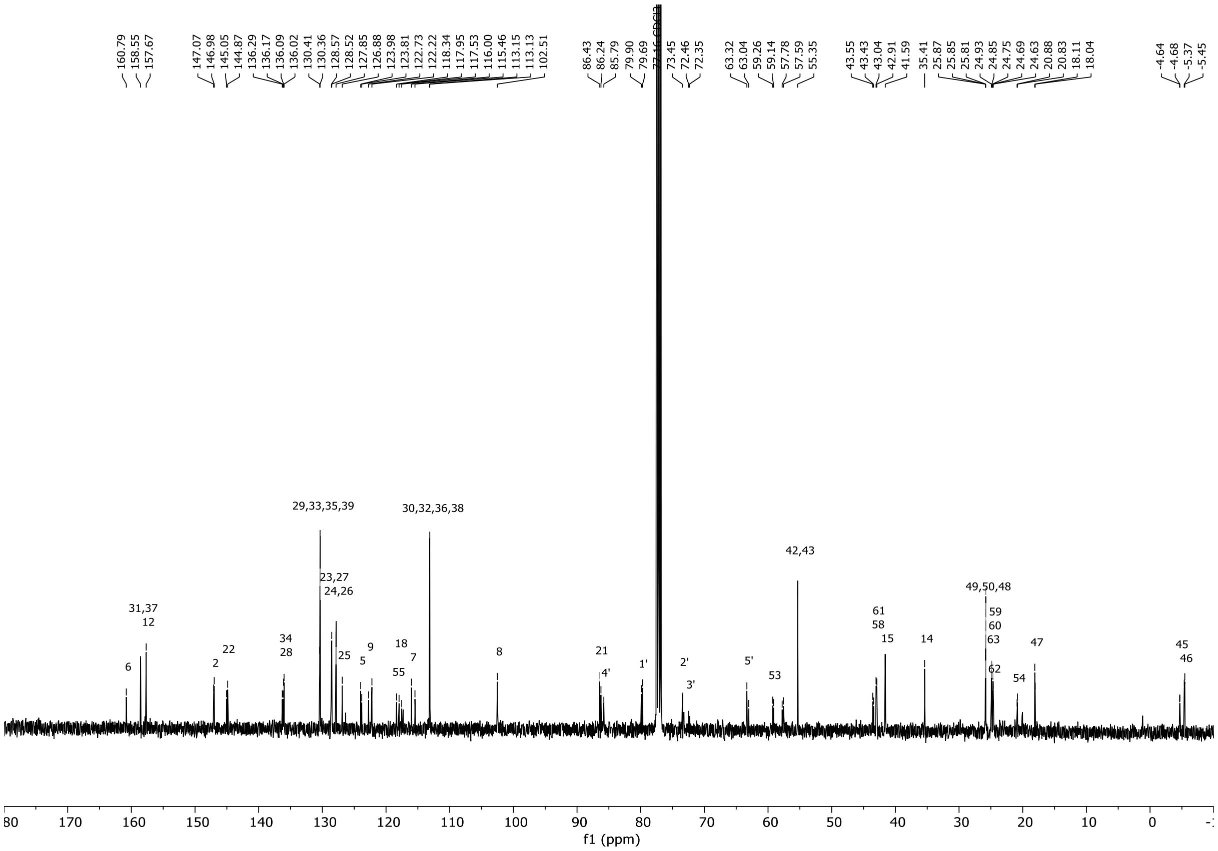

^{13}C NMR (100 MHz, $CDCl_3$) of compound **5**.

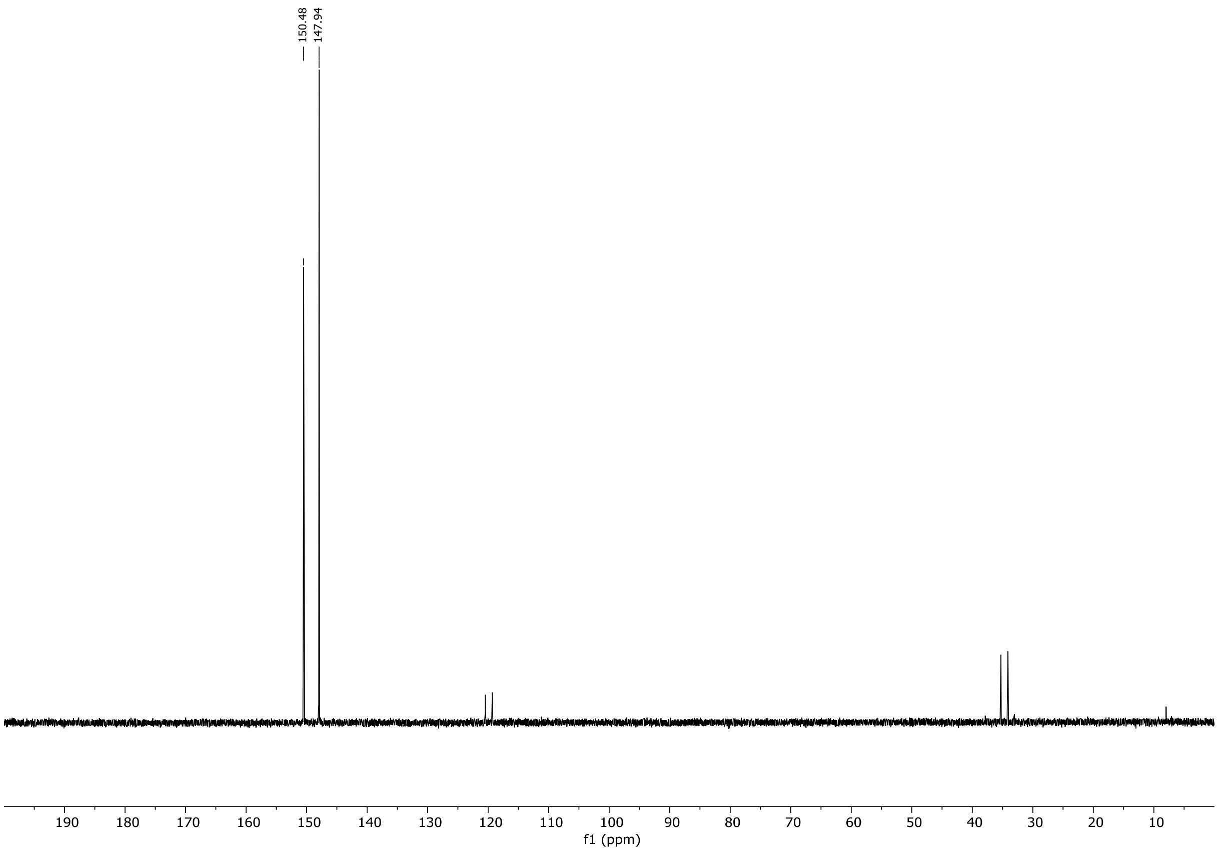

^{31}P NMR (162 MHz, $CDCl_3$) of compound **5**.

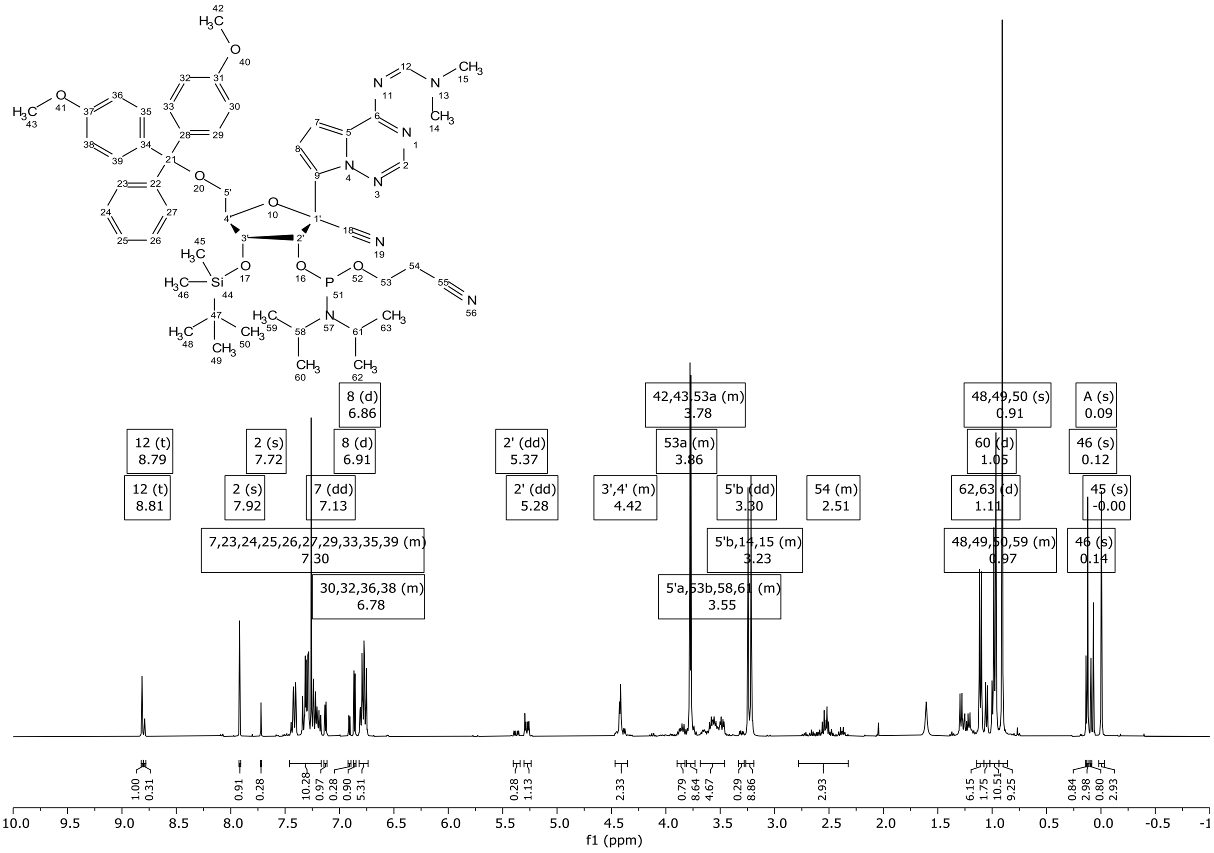

^{1}H NMR (400 MHz, $CDCl_3$) of compound **6**.

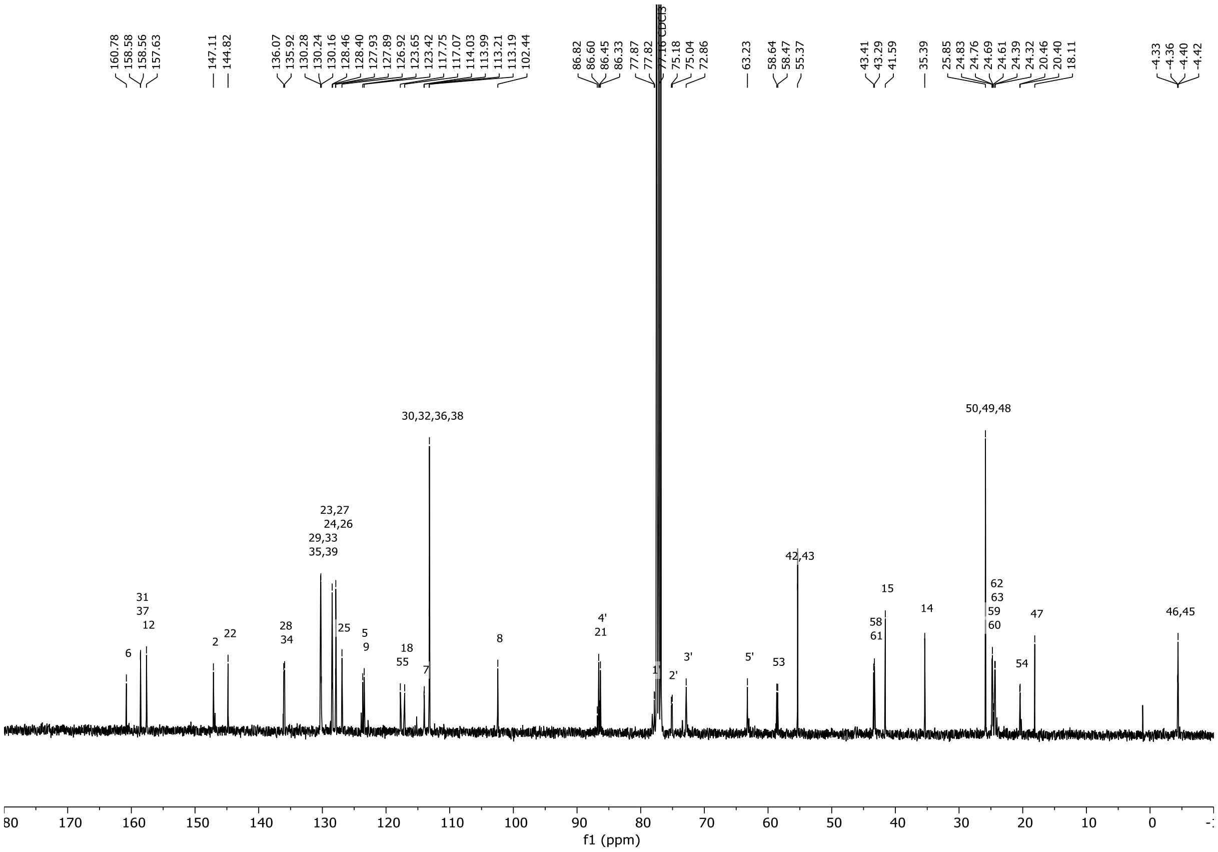

^{13}C NMR (100 MHz, $CDCl_3$) of compound **6**.

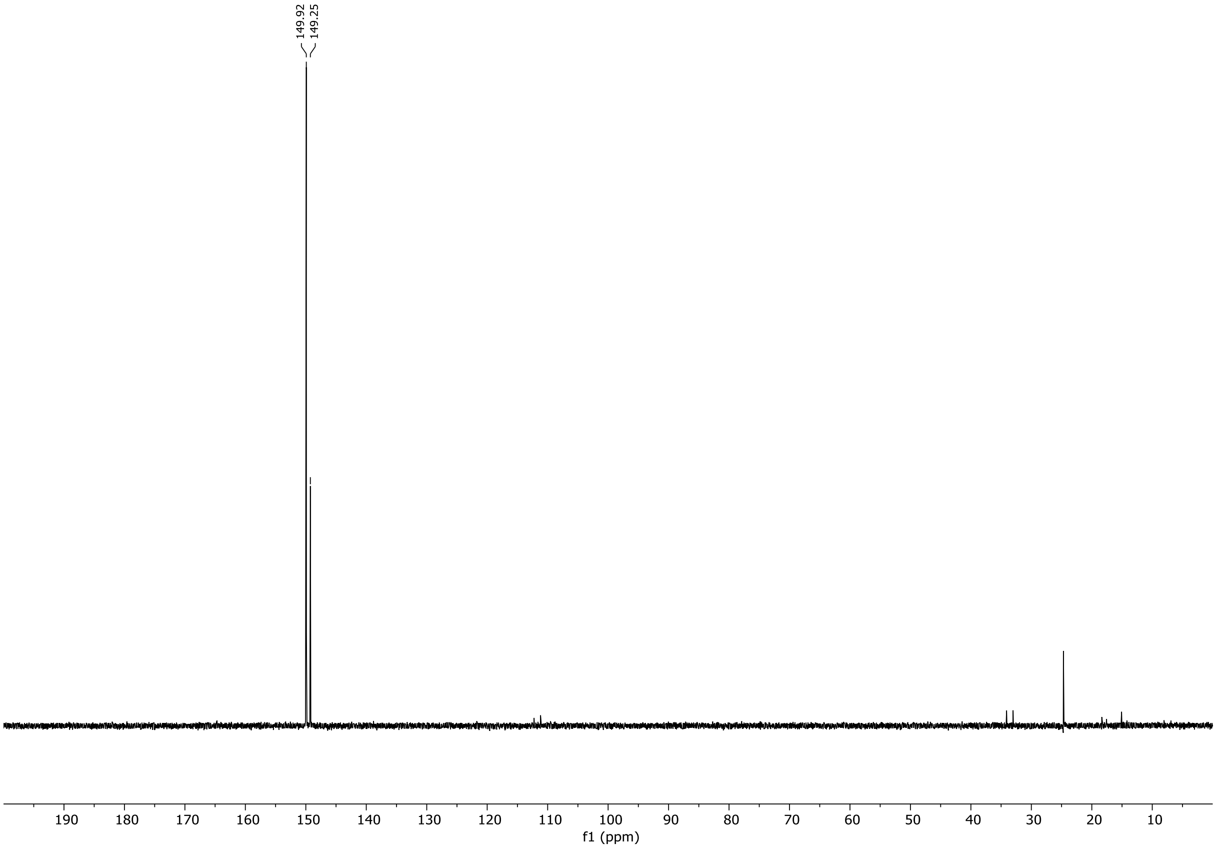

^{31}P NMR (162 MHz, $CDCl_3$) of compound **6**.

Supplementary References

[1] a) Caton-Williams, J.; Smith, M.; Carrasco, N.; Huang, Z. Protection-free one-pot synthesis of 2′-deoxynucleoside 5′-triphosphates and DNA polymerization. *Org. Lett.* **2011**, *13*, 4156-4159; b) Caton-Williams, J.; Hoxhaj, R.; Fiaz, B.; Huang, Z. Use of a Novel 5′-Regioselective Phosphitylating Reagent for One-Pot Synthesis of Nucleoside 5′-Triphosphates from Unprotected Nucleosides. *Curr Protoc Nucleic Acid Chem.* **2013**, *52*, 1.30.1 – 1.30.21, doi:10.1002/0471142700.nc0130s52.

[2] Warren, T. K.; Jordan, R.; Lo, M. K.; Ray, A. S.; Mackman, R. L.; et al. Therapeutic Efficacy of the Small Molecule GS-5734 Against Ebola Virus in Rhesus Monkeys. *Nature* **2016**, *531*, 381-385.
