# Supplementary material for: Mechanism of SARS-CoV-2 polymerase stalling by remdesivir
Source: Nat Commun. 2021 Jan 12;12:279. doi: 10.1038/s41467-020-20542-0 (PMC7804290; doi:10.1038/s41467-020-20542-0)
Supplement: Supplementary file 3 — Description of Additional Supplementary Files [file 41467_2020_20542_MOESM3_ESM.pdf]

Description of Additional Supplementary Files

Supplementary Movie 1: Animation of remdesivir-induced RdRp stalling

Description: Remdesivir (purple, C1'-cyano group in van der Waals surface rendering) binds to the +1 site and base pairs with a uridine in the template strand (blue) and is then incorporated into the growing RNA product (light red). For simplicity, NTP binding and release of pyrophosphate were omitted and instead only the RMP moiety is shown as it is added to RNA. Translocation of the RNA then transfers the RNA product 3'-nucleotide from position +1 to position –1. Such nucleotide addition cycle is repeated three more times using cognate, natural nucleotides (red). After addition of the third nucleotide, translocation is impaired because the C1'-cyano group of remdesivir encounters the side chain of serine-861 (shown in van der Waals surface rendering) of RdRp subunit nsp12. The translocation barrier is indicated by failed attempts to translocation that are shown twice for clarity. Note that unknown structural adaptations allow for passage of the translocation barrier (not shown). Location of the RdRp active site is indicated by a magenta sphere.
