# Supplementary material for: Mechanism of SARS-CoV-2 polymerase stalling by remdesivir
Source: Nat Commun. 2021 Jan 12;12:279. doi: 10.1038/s41467-020-20542-0 (PMC7804290; doi:10.1038/s41467-020-20542-0)
Supplement: Supplementary file 2 — Peer Review File [file 41467_2020_20542_MOESM2_ESM.pdf]

Peer Review Information

Editorial Note: This manuscript has been previously reviewed at another journal that is not operating a transparent peer review scheme. The manuscript was considered suitable for publication without further review at *Nature Communications*.
